# Affirmative action programs and network benefits in the number of board positions

**DOI:** 10.1371/journal.pone.0236721

**Published:** 2020-08-04

**Authors:** Katarzyna Burzynska, Gabriela Contreras

**Affiliations:** 1 Institute for Management Research, Radboud University, AJ Nijmegen, The Netherlands; 2 Knut Wicksell Centre for Financial Studies, Lund University, Lund, Sweden; The Bucharest University of Economic Studies, ROMANIA

## Abstract

Whereas governments are increasingly considering affirmative action programs to increase corporate board diversity, the effect of such programs can be superficial as they do not address the underlying problem, which is women’s access to and inclusion in relevant corporate networks. To address this issue, we study the relationship among affirmative action programs (binding gender quotas and non-binding gender targets), director networks, and the number of board positions individual directors hold given their gender. We use personal, professional, and network characteristics of 25,127 unique directors from 2,435 public firms in 32 European countries over the period of 2000 through 2017. We find that in the absence of affirmative action programs, women directors benefit less from their networks than men directors suggesting the existence of a gender gap in network benefits. After the passage of binding gender quotas, this gender gap in network benefits narrows between women and men directors. Overall, this research suggests that binding gender quotas make director networks a more salient tool for hiring women and may help in leveling the playing field in the way these networks are used for achieving top management positions.

## Introduction

“I thought General [George C.] Marshall might do us some good […] recognizing the position he holds in the community […] and the acquaintances he has” wrote Alfred P. Sloan –board chair at General Motors– in a letter to Lammot du Pont –a major shareholder of General Motors– in 1945 [1]. Alfred P. Sloan’s recommendation of General Marshall on the basis of his network was and still is a prevalent practice in hiring board members. These hiring practices, however, are not generally inclusive because men within the same networks tend to choose one another likely eliminating most women [2]. In fact, as of 2018, women directors occupied only 20% of boards positions worldwide [3]. This women’s underrepresentation in boards has raised both ethical and economic concerns triggering the public interest and some governments to stimulate equal opportunity in board representation. Yet the effect of government intervention in increasing board diversity can be superficial, if any, as it does not address an underlying problem, which is women’s access to and inclusion in relevant corporate networks.

While crucial in career advancement –for example, in obtaining earlier promotions [4], better job evaluations [5], and access to influential others [6]– networks often times may be disadvantageous to women. Gender barriers in network formation not only can lead to differences in the composition of the networks being formed but also can lead to differences in the importance of the position held within these networks [7–9]. Additionally, similar networks can offer different benefits based on gender with women typically benefiting less than men [10–13].

Ample evidence from qualitative studies about board appointments and gender disparity highlights that board positions are filled through “shoulder tapping” among acquaintances and colleagues [14, 15], where CEOs and standing board members nominate candidates for board membership [16]. This prevalent reliance on networks, though, may contribute to women’s underrepresentation in boards. To illustrate, interviews among directors suggest the existence of an “old boys’ network” of corporate directors where a “who-you-know” approach to recruiting immediately eliminates most women candidates [2]. Compared to interview-based studies about board appointments and gender disparity, quantitative studies in the same area have largely ignored the role of networks and the potential network benefits directors can reap depending on their gender and rather focused on the role of human capital [17–19], firm [17, 20, 21], industry [19, 22, 23], and country characteristics [19, 24, 25].

One way to reduce the gender disparity in board representation is through affirmative action programs. Affirmative action is a “generic term for policies aimed at encouraging and supporting under-represented groups within a workplace” [26, pp.729]. In Europe, two affirmative action programs to stimulate board diversity include binding gender quotas and non-binding gender targets. Binding gender quotas are a form of hard law requiring public firms to meet gender diversity requirements. If the gender diversity requirements are not met, firms face sanctions. In contrast, non-binding gender targets are a form of soft law under which firms may meet gender diversity targets. However, if the gender diversity targets are not met, firms do not face any sanctions. Because networks contribute to gender disparity in board representation, when evaluating the impact of affirmative action programs, it is crucial to better understand whether and how these programs moderate the role of networks in the number of board positions held by both men and women directors.

Despite the importance of networks in perpetuating gender disparities in board representation, the question of whether and to what extent affirmative action programs play a role in alleviating these gender differences has thus far received no attention in the literature. Closest to our paper are [27, 28] and [29]. [27] compare the social capital –derived from network positions– of men and women Norwegian directors holding multiple board positions at public firms before and after the introduction of a binding gender quota in Norway. [28] and [29] compare how different types of social capital in a sub-sample of *prominent* women directors holding multiple board positions at public firms change before and after the introduction of binding gender quotas in Norway and Italy, respectively. However, to the best of our knowledge there is no other research addressing the gender difference in the benefits –in terms of board positions– directors may derive from their social networks and whether the passage of different types of affirmative action programs plays a role in alleviating these gender differences. As such, this is the first cross-country study examining how the passage of affirmative action programs may enhance the inclusion of women directors by changing the role networks have had in perpetuating gender disparities in board representation.

Here, we shed light on the relationship between affirmative action programs, director networks, and the number of board positions directors hold. The recruitment process for board directors works predominantly through nominations by standing directors and CEOs [16]. Therefore, those with higher social capital, that is, those with ties to important members in a network of board members at institutions such as clubs, military, charitable, government, sporting, educational, and medical as well as in public and private companies worldwide have a better chance at having their names brought forward when board positions are awarded. A statement from a board chair at a Dutch company further illustrates this practice: “We look in our own networks […] You can not just bring in a complete stranger for an important position” [30, p.500]. According to social identity theory, the gender composition of those who recruit directors, i.e. the recruiting committees consisting of standing directors and the CEO, is pivotal in who gets the board positions. Because recruiting directors are mostly men, they are more prone to choose in-group members –that is, men candidates. Whereas women, being out-group members, are less likely to be treated on an equal basis with men even when having the same qualifications and social capital. Instead, women tend to be seen as too different from standing board members who value homogeneity and group cohesiveness [31]. Consequently, women tend to benefit less than men from the same level of social capital and would need to engage in higher level of social influence behavior to have the same opportunities as men in obtaining board positions [32].

By setting more explicit requirements about gender representation in boards, we expect affirmative action programs to affect the director selection process as follows. Due to the need to increase women’s representation in boards, selection committee members are expected to look more actively for women in their networks. Since there is a more active search for women candidates, women are likely to become more visible with this visibility likely to become more comparable to that of men. As the visibility of women becomes more equal to that of men, women cease to be seen as the “other” and the influence of in-group/out-group biases lessen [33]. Therefore, we expect women to benefit more from their networks after the passage of affirmative action programs. However, these changes may likely depend on whether the affirmative action program is binding or not.

The contributions of this research are fivefold. First, different types of affirmative action programs have not been assessed as potential factors determining the extent to which network benefits in board positions vary by gender. Previous network studies in career advancement focus on in-group/out-group biases [4, 10–12] showing that women benefit less than men from their networks. We extend the knowledge on the relationship between networks, gender, and career advancement to settings where there are changes in the legal environment, i.e. passage of affirmative action programs. Moreover, we distinguish between different affirmative action programs, namely binding gender quotas and non-binding gender targets. As such, this research enhances our understanding of the consequences of affirmative action programs, and responds to the frequent requests to assess these programs beyond their formal fulfillment [19, 34–36].

Second, we add to the mostly descriptive studies on the social capital characteristics of directors, both in terms of data and statistical approaches. In terms of data, we extend the knowledge of existing studies which have focused mainly on individual countries (for example, the Netherlands [37], Norway [27, 28], and Italy [29]) and on a selection of a few, prominent directors, by using a cross-country sample of 25,127 unique directors –regardless of prominence– from 2,435 public firms in 32 European countries over the period of 2000 through 2017. In terms of statistical approaches, we use a battery of statistical methods including multi-level linear models and matching techniques. As such, our research contributes to the call for more empirical and cross-country research on board gender diversity and affirmative action programs [36].

Third, we use more comprehensive information to capture the social capital of directors when constructing their social networks. Specifically, instead of exclusively relying on information from board interlocks between directors at listed firms, we identify the social capital of directors stemming from their board positions at institutions such as clubs, military, charitable, government, sporting, educational, and medical as well as in public and private companies worldwide. Since ties among directors may be formed outside the boardrooms of public firms [38], our approach allows us to get a broader representation of the underlying social network among directors than the networks of corporate interlocks –at public firms only– often used in the literature.

Fourth, this study adds to a stream of literature that extends beyond the business case for gender diversity. Experimental research provides insights into the effects of affirmative action programs on the incentives for the disadvantaged minorities to enter competitive environments, e.g. [39–43], on the reduction of gender discrimination in hiring decisions, e.g. [44], and on the enhancing of women’s labor market participation, e.g. [43–47]. However, research particularly addressing the effects of binding gender quotas on women representation in corporate boards has been dominated by the business case approach, i.e. has focused on firm-level performance. See for example, [48–55]. As a result, much less is known about the consequences gender quotas may have on individual directors as gender equality policies are implemented, especially considering that board positions are obtained through social networks. Our study seeks to further this understanding.

Last, our paper contributes to important and current international debates regarding the adoption of affirmative action programs to stimulate gender diversity in board representation. While governments are increasingly considering to adopt affirmative action programs to increase board diversity, the effect of these interventions can be superficial as they do not address the underlying problem, which is women’s access to and inclusion in relevant corporate networks. We address this problem by specifically considering whether and to what extent the passage of different types of affirmative action programs changes the benefits directors extract from their networks. As such, our research provides grounds for a better understanding of the direct and indirect implications associated with different types affirmative action programs: binding gender quotas and non-binding gender targets.

## Data and methods

### Data

We study the link between affirmative action programs, director networks, and the number of board positions directors hold by leveraging a large data set of individual directors in European public firms over the period of 2000 and 2017.

Our data comes from different sources. Information about directors, such as their board positions (in clubs, military, charitable, government, sporting, educational, and medical institutions as well as in public and private companies worldwide), their professional experience, and their educational background comes from BoardEx [56]. BoardEx is a commonly used commercial database in studies about board composition, see for example [57–59]. Financial information about the firms where our directors sit comes from Thomson Reuters Datastream [60]. Last, information about affirmative action programs (binding gender quotas or non-binding gender targets) and their corresponding passage years are obtained from [33, 36, 61, 62]. S1 Table in the S2 Appendix lists all data sources for the different measures used in this research.

**Fig 1:**
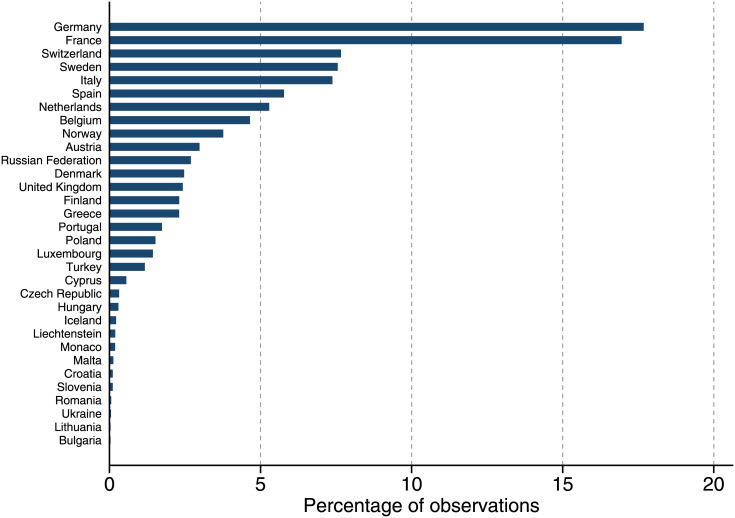
Geographical distribution of our sample of directors. The directors in our sample sit in boards across 32 different European countries. Most of the directors in our sample sit in German and French boards accounting for 34% of our data.

Our sample is constructed as follows. First, we exclude those directors with missing director-level and firm-level characteristics. Second, to be able to study the effect of country-wide affirmative action programs, we also exclude 890 directors having board positions at companies in multiple countries in a given year. These 890 directors represent only 3.4% of all observations. The final sample contains 25,127 unique directors, from 2,435 public firms in 32 European countries over the period of 2000 through 2017. Fig 1 displays the geographical distribution of our observations. Most of the directors in our sample sit in German and French boards accounting for 34% of our data. See S2 Appendix for more details on how we construct our sample.

**Fig 2:**
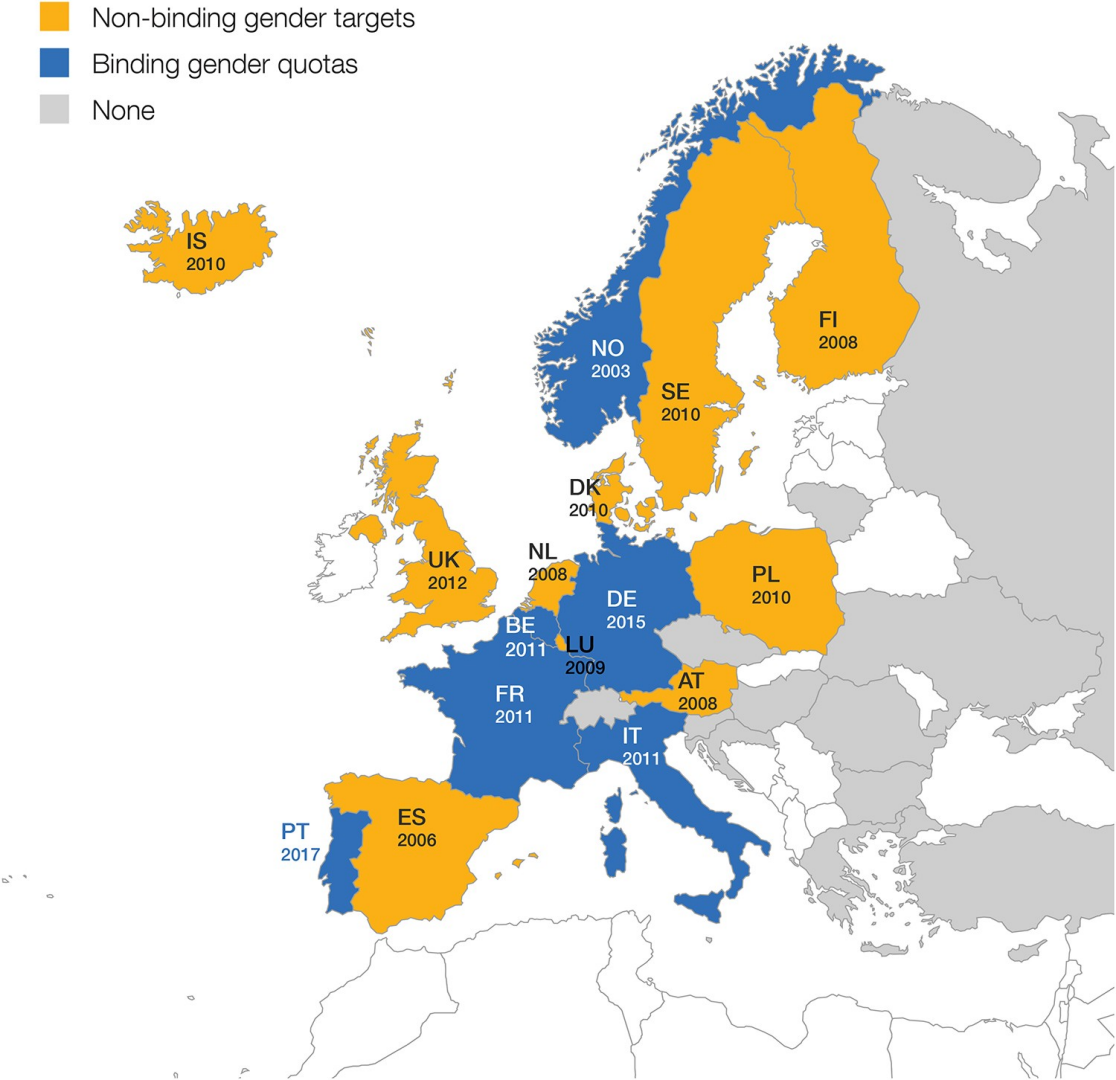
Map displaying the two types of affirmative action programs in the 32 European countries our sample of directors comes from. This map shows the latest affirmative action program passed in each country and the corresponding passage year. While some countries that have passed non-binding gender targets switched to binding gender quotas (Germany, Belgium and France), for illustration purposes we only present the latest affirmative action program passed in each country. According to this criteria, out of the 32 countries where our sample of directors hold board seats, a total of six have passed binding gender quotas (in blue), while ten have passed non-binding gender targets (in yellow). The remaining 17 countries (in gray) have not passed either binding gender quotas or non-binding gender targets during our observation period of 2000 and 2017. The data about the affirmative action program type and corresponding passage years was collected from: [33, 36, 61, 62]. Countries not included in our sample are in white.

Out of the 32 countries the directors in our sample come from, 16 have introduced affirmative action programs. Fig 2 shows the countries that have passed affirmative action programs and the corresponding passage year. Among these countries, six have introduced binding gender quotas. The first country to pass a binding gender quota was Norway in 2003, followed by Italy, Belgium, and France in 2011, Germany in 2015, and Portugal in 2017. Ten other countries have introduced non-binding gender targets between 2008 and 2012. Note that Germany, Belgium and France first passed non-binding gender targets and then switched to binding gender quotas. The binding gender quotas in our study are a form of hard law requiring public firms to meet gender diversity requirements. If the gender diversity requirements are not met, firms face sanctions. The non-binding gender targets in our study are a form of soft laws under which public firms may meet gender diversity targets. If the gender diversity targets are not met, firms do not face any sanctions. In terms of the gender diversity goals the affirmative action programs seek to attain, we note that the binding gender quotas vary between 20% (Portugal) and 40% (France and Norway), with an average of 33% of women representation in boards. A third of the non-binding gender targets recommend firms to reach between 20% (France) and 40% (Spain and Iceland) woman representation in boards with an average of 33%. The remaining non-binding gender targets recommend firms to have both genders represented in boards without indicating a specific share of woman representation. For a more detailed overview of our binding gender quotas and non-binding gender targets, see Table 1.

**Table 1:** Affirmative action programs applicable to the 32 European countries our sample of directors comes from. The binding gender quotas in our study are a form of hard law requiring public firms to meet gender diversity requirements. If the gender diversity requirements are not met, firms face sanctions. The non-binding gender targets in our study are a form of soft laws under which public firms may meet gender diversity targets. If the gender diversity targets are not met, firms do not face any sanctions. Columns *Quota* % in Panel A and *Target* % in Panel B report the percentage of board gender representation laid out by the affirmative action program. *n.s*. indicates that a percentage of gender representation has not been specified. *Passage year* indicates the year when the affirmative action program was passed. The column labeled *Notes* provides information on sanctions for binding gender quotas (Panel A), and the recommendations for non-binding gender targets (Panel B). Note that Germany, Belgium and France first passed non-binding gender targets and then switched to binding gender quotas.

Panel A: Binding gender quotas			
Country	Quota %	Passage Year	Notes
Norway	40%	2003	Sanctions include refusal to register the board, firm dissolution, and fines if non-compliant. Source: [33].
Italy	33%	2011	Sanctions include fines and directors losing office. Source: [33]
France	40%	2011	Sanctions include fees not being paid to directors. Source: [33, 36]
Belgium	33%	2011	Sanctions include suspension of benefits and compensation for all board members. Source: [33, 36]
Germany	30%	2015	Sanctions include leaving director seat vacant. Source: [33]
Portugal	20%	2017	Sanctions include fines. Source: [62]
Panel B: Non-binding gender targets			
Country	Target %	Passage Year	Notes
Spain	40%	2007	No sanctions but the extent to which public subsidies and contracts are given depends on board diversity. Source: [33, 36].
Austria	n.s.	2008	Specific gender diversity target set out for state-owned enterprises only. Source: [36].
Belgium	30%	2008	Source: [36].
Finland	n.s.	2008	Both genders need to be present on the board. Source: [36].
Netherlands	n.s.	2008	Targets are determined by the companies themselves. As of 2011, a target of 30% for public firms with more than 250 employees was set out. Source: [36, 61].
Luxembourg	n.s.	2009	Both genders need to be present on the board. Source: [61].
Denmark	n.s.	2010	According to the Danish Corporate Governance code, firms should evaluate the selection of candidates in light of gender diversity. Source: [61].
France	20%	2010	For smaller boards of fewer than nine directors, the difference between genders should exceed two. Whenever a board does not have any women directors upon the release of the annual report, they should nominate one by the second general meeting. Source: [36].
Germany	n.s.	2010	Boards should consider gender diversity when appointing directors to its management board. Source: [36].
Iceland	40%	2010	Applicable to firms with more than 50 employees. Source: [33].
Poland	n.s.	2010	A balanced proportion of both genders needs to be present on the board. Source: [61].
Sweden	n.s.	2010	A balanced proportion of both genders needs to be present on the board. Prior to 2010, firms had to disclose their gender breakdown according to a disclosure rule established in 2007. Source: [61].
United Kingdom	n.s.*	2012	According to the UK Corporate Governance code, firms should evaluate their boards in light of gender diversity. Source: [36, 61].

The variables of interest in our analyses are defined as follows. First, the dependent variable is the number of board positions a director holds at public firms in a given year. As we study affirmative action programs in Europe that are applicable to public firms only, our dependent variable measures the number of board positions held at European public firms, see S1 Appendix. Using the number of board positions as a dependent variable allows us to focus on the extent to which directors participate in corporate decision-making by holding board positions.

Second, we characterize a director’s network with the variable eigenvector centrality. Calculating eigenvector centrality requires us to map the network of directors. To do this, we collect information on the board positions directors hold in clubs, military, charitable, government, sporting, educational, and medical institutions as well as in public and private companies worldwide. In the network, a pair of directors *i* and *j* is defined as tied if both sit on the same board in the same year. Sitting on boards allows directors to build a network of acquaintances and colleagues that can potentially serve as an outreach mechanism for hiring committees. While the way in which we operationalize our networks may not offer a full representation of the real underlying social network among directors, our networks are broader than the networks of corporate interlocks often used in the literature that are constructed from directors’ board positions at public firms only, for example [37].

Eigenvector centrality is a measure that comes from the social networks field. It is a commonly used measure to capture social capital where social capital stems from connections actors have with one another [63, 64]. In our setting, this translates to the connections directors have with one another. Eigenvector centrality captures the importance of a director’s social capital in the sense that a director with a high eigenvector centrality is connected to directors that themselves are well connected to other directors that themselves are well connected [64]. In this sense, an important member in the network is somebody who is connected to well-connected others.

Using eigenvector centrality allows us to consider a director’s direct and indirect ties, thus taking into account the entire pattern in the network. This measure is particularly applicable in our context because there is high variation in the importance of the position directors have within their networks. To exemplify, some directors are connected to directors with few ties (few indirect ties), while some other directors are connected to directors with many ties (many indirect ties). A director with many ties to peripheral directors having few ties would have a lower social capital than directors with few ties to highly connected directors. We choose eigenvector centrality over other graph-theoretic measures like degree centrality because these other measures would not allow us to capture the different patterns in direct and indirect ties or the varying levels in social capital [65]. That is, eigenvector centrality allows us to capture the degree to which directors have relations with important members in the network.

It is important to clarify that eigenvector centrality differs from our dependent variable (number of board positions held at European public firms) both theoretically and empirically. Theoretically, eigenvector centrality does not only capture the importance of direct ties which may be related to the number of board positions held at European public firms, but also the importance of indirect ties which are not related to the number of board positions held. Empirically, eigenvector centrality captures the importance of a director’s social capital stemming for their board positions at various institutions –besides those at European public firms– and has a low correlation with our dependent variable (*ρ* = 0.219), which captures the number of board positions at European public firms only (see Table S4 in S3 Appendix). Moreover, consistent with other longitudinal network studies, we lag the eigenvector centrality score, see for example [66].

Given the large number of directors in our sample, directors typically have ties to only a small portion of the directors in the network. As a result, the average director in our sample has an eigenvector centrality equal to 0.81 on a standardized scale between zero and 100, see S3 Table in S3 Appendix. These relatively small eigenvector centrality scores are consistent with other studies of director networks, see for example [66].

Third, we identify a director’s gender with a dummy variable that is equal to one when the director is a woman and zero otherwise. Our sample consists of mostly men directors (88%).

Last, we classify countries having affirmative action programs into two categories: binding gender quotas and non-binding gender targets. Based on this classification we create two different dummy variables, one for each affirmative action program type. The detailed definitions of all the variables used in our analyses, including control variables, can be found in S1 Appendix.

### Statistical analysis

We study the link between affirmative action programs, director networks, and the number of board positions directors hold at European public firms. By focusing on Europe, we exploit the statistical properties of a quasi-natural experiment. Specifically, the passage of affirmative action programs concerning gender diversity in Europe allows us to exploit exogenous variation in our explanatory variables [67, 68]. Since different forms of affirmative action programs (binding gender quotas and non-binding gender targets) have been passed at different points in time across different countries in Europe, we are able to observe the number of board positions directors hold before and after the passage of these affirmative action programs while having some directors not being subject to any, i.e. not being exposed to treatment effects.

Since directors (individual level) may hold board positions at multiple firms within the same country, they are nested at the country level (contextual level). Therefore, our data is hierarchical with two levels. In order to account for this hierarchical structure, we estimate a series of multi-level regressions [69]. With these regressions, we investigate the relationship between gender, networks, affirmative action programs, and the number of board positions directors hold. Specifically, our main variable of interest is an interaction term between gender, networks, and the affirmative action program. Because we observe two forms of affirmative action programs, binding gender quotas and non-binding gender targets, we fit two separate models –one for each form of affirmative action program. We fit two separate models for ease of interpretation. In robustness analysis, we also estimate one model with a three-level categorical variable accounting for binding gender quotas, non-binding gender targets, and no affirmative action program yielding consistent results. See S5 Appendix. Specifically, the binding gender quota model includes countries that either have passed binding gender quotas (including those that have switched from non-binding gender targets to binding gender quotas) or have not passed any form of affirmative action program. The non-binding gender target model includes countries that either have passed non-binding gender targets (including those that have later switched from non-binding gender targets to binding gender quotas) or have not passed any form of affirmative action program. In both models, we control for lagged director, firm, industry, country, and network characteristics and we also account for countries switching from non-binding gender targets to binding gender quotas in our analyses. As we conduct our analysis at the director level, we aggregate firm-specific information for each director (see S1 Appendix).

We further conduct a series of additional tests using quasi-experimental methods (entropy balanced matching and coarsened-exact matching (CEM)), different estimation approaches (random effects panel data with year and country fixed effects and panel Poisson regressions), and time-lagged analysis, see Additional tests. In general, the results of all the additional tests are consistent with the results of the main analysis.

## Results

Table 2 presents the results from two multi-level regressions. In the first regression, we estimate the relationship between gender, networks, binding gender quotas, and the number of board positions. In the second regression, we estimate the relationship between gender, networks, non-binding gender targets, and the number of board positions.

**Table 2:** Multi-level regression of number of board positions on director’s gender, networks and affirmative action program types.

	Affirmative action program	
	Binding gender quota	Non-binding gender target
	(1)	(2)
Woman director × Affirmative action program × Eigenvector centrality	0.028***	0.001
	[0.006]	[0.003]
Woman director × Affirmative action program	0.049***	0.021
	[0.016]	[0.018]
Affirmative action program × Eigenvector centrality	-0.013***	-0.011***
	[0.002]	[0.001]
Woman director × Eigenvector centrality	-0.018***	-0.014***
	[0.002]	[0.002]
Woman director	-0.040***	-0.028***
	[0.012]	[0.009]
Eigenvector centrality	0.037***	0.039***
	[0.001]	[0.001]
Affirmative action program	0.018**	-0.018***
	[0.007]	[0.007]
*Control variables*		
Age	0.016***	0.015***
	[0.002]	[0.002]
Age^2^	-0.000***	-0.000***
	[0.000]	[0.000]
Graduate degree	0.064***	0.061***
	[0.005]	[0.004]
Board experience	0.652***	0.659***
	[0.007]	[0.006]
Maximum firm size	0.026***	0.028***
	[0.001]	[0.001]
Maximum firm profitability	0.453***	0.484***
	[0.023]	[0.021]
Large component	0.065***	0.064***
	[0.010]	[0.009]
Small board size sector	-0.261***	-0.252***
	[0.006]	[0.005]
Country’s stock market size (%)	0.070**	0.069**
	[0.033]	[0.030]
Affirmative action program switch	0.010	0.005
	[0.013]	[0.012]
Constant	0.015	-0.008
	[0.135]	[0.132]
HLM estimated SD (constant)	0.090***	0.108***
	[0.019]	[0.017]
HLM estimated SD (residual)	0.653***	0.651***
	[0.002]	[0.002]
Observations	74289	88402
LR Test	26444.145	29980.224
Log-likelihood	-73830.571	-87569.161

Standard errors in brackets

* *p* < 0.10,

** *p* < 0.05,

*** *p* < 0.010

**Fig 3:**
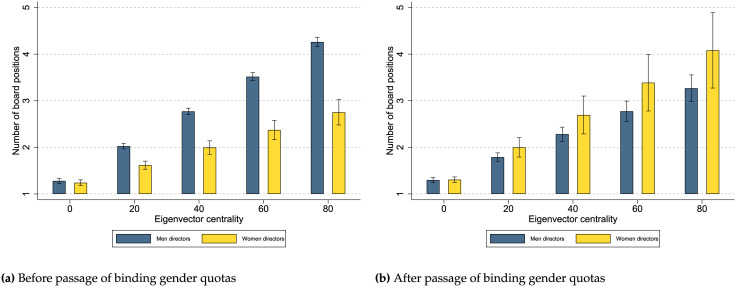
Relationship between gender and network benefits before and after the passage of binding gender quotas. The passage of binding gender quotas is associated with a change in the benefits men and women directors extract from their networks: after the passage of binding gender quotas, networks become more beneficial for women than before and more similar to the network benefits of men directors when it comes to attaining board positions. Margin plots are estimated from the coefficients reported in Table 2. These figures show that directors with a higher eigenvector centrality are associated with more board positions. (a) indicates that men directors benefit more from their networks than women directors before the passage of any binding gender quotas. However, (b) indicates that the network benefits between women and men directors are not statistically different after the passage of binding gender quotas.

Our main results show that the way women directors benefit from networks varies depending on whether or not affirmative action programs are in place, as well as whether the affirmative action program is binding or non-binding. The passage of binding gender quotas is associated with an increase in network benefits for women directors as indicated by the positive and statistically significant coefficient for the interaction term *Woman director × Binding gender quota × Eigenvector centrality*. Fig 3 presents the margin plots with 95% confidence intervals for the predicted number of board positions women and men directors obtain depending on their networks and if there are not any binding gender quotas (Fig 3a) or if there are binding gender quotas (Fig 3b). In general, Fig 3 shows that the gender differences in network benefits vary depending on the presence of binding gender quotas. Specifically, Fig 3a shows that women directors benefit less from their networks than men directors when there are not any binding gender quotas. However, in the presence of binding gender quotas (Fig 3b), women directors benefit more from their networks than in the absence of binding gender quotas. The increase in network benefits women directors gain after quotas results in women and men directors reaping more similar benefits from their networks making any gender differences statistically insignificant once binding gender quotas are passed.

**Fig 4:**
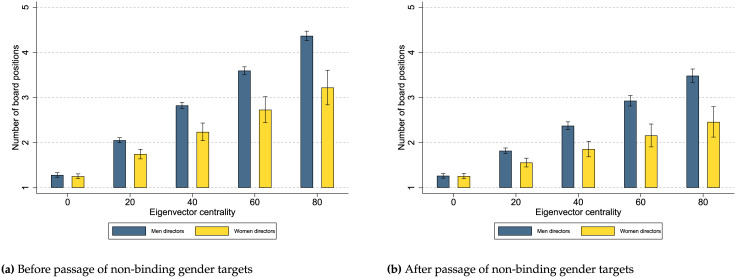
Relationship between gender and network benefits before and after the passage of non-binding gender targets. In contrast to Fig 3, passage of non-binding gender targets is not associated with a change in the benefits men and women directors extract from their networks: in the presence of binding gender quotas, networks remain more beneficial for men than for women directors in attaining board positions. Margin plots are estimated from the coefficients reported in Table 2. These figures show that directors with a higher eigenvector centrality are associated with more board positions. Both (a) and (b) indicate that men directors benefit more from their networks than women directors regardless of the passage of non-binding gender targets.

In contrast to binding gender quotas, the passage of non-binding gender targets does not change the network benefits women directors reap as the statistically insignificant coefficient for the interaction term *Woman director × Non-binding gender target × Eigenvector centrality* in Table 2 shows. Fig 4 presents the margin plots with 95% confidence intervals for the predicted number of board positions women and men directors obtain depending on their networks and if there are not any non-binding gender targets (Fig 4a) or if there are non-binding gender targets (Fig 4b). In general, Fig 4 indicates that network benefits for women directors do not depend on the passage of non-binding gender targets. Specifically, comparing Fig 4a and Fig 4a indicates that network benefits are higher for men directors than for women directors regardless of the passage of non-binding gender targets.

Overall, we find that in the absence of affirmative action programs (Figs 3a and 4a), women directors benefit less from their networks, than men directors, suggesting the existence of a gender gap in network benefits. Only in the case of binding gender quotas, this gender gap in network benefits narrows between women and men directors (Figs 3b and 4b). We should note that the statistical differences between the estimated coefficients across the two regressions are not formally tested; however, we estimate another model with a three-level categorical variable for *Affirmative action program* that allows us to compare the statistical differences of the estimated coefficients with results remaining robust to those presented in our main regressions. See S5 Appendix. Our results indicate that binding gender quotas are more efficient than non-binding gender targets in increasing the network benefits for women directors possibly leading to a more inclusive board appointment process.

To further complement our results, we compute and plot the marginal effects for the variables eigenvector centrality, woman director, and affirmative action program. See S2 Fig and S3 Fig in S4 Appendix. Because these variables are all part of our interaction term, we evaluate the marginal effects by setting the other variables in the interaction equal to different values (i.e. zero or one for woman director; zero or one for affirmative action program; zero, 20, 40, 60 or 80 for eigenvector centrality) and holding all control variables equal to their mean.

We note the following. First, for both women and men directors, the marginal effects of eigenvector centrality for the number of board positions are positive and statistically significant before and after the passage of binding gender quotas and non-binding gender targets. This result is in line with the general notion that networks are important in career advancement. Second, for all values of eigenvector centrality, the marginal effects of woman director for the number of board positions are positive after the passage of binding gender quotas and negative before the passage of binding gender quotas; whereas, these marginal effects are negative both before and after the passage of non-binding gender targets. This implies that, irrespective of the value of eigenvector centrality, women directors, with respect to men, experience an increase in the number of boards they hold after the passage of binding gender quotas; whereas irrespective of the value of eigenvector centrality, women hold fewer board positions than men directors both before and after the passage of non-binding gender targets. Third, for all values of eigenvector centrality, the marginal effects of the passage of binding gender quotas are positive for women directors and negative for men directors. While, for all values of eigenvector centrality, the marginal effects of the passage of non-binding gender targets are negative for both women directors and men directors. In general, the passage of binding gender quotas is associated with a positive and significant change in the number of board positions women directors hold while the passage of non-binding gender targets is not. This finding suggests that binding gender quotas are more successful than non-binding gender targets in increasing the number of board positions women directors hold. This finding is in line with recent evidence that clear enforcement mechanisms such as binding gender quotas are more successful in increasing women representation on boards than non-binding gender targets [70].

Examining the relations between our control variables and the number of board positions, Table 2 indicates that older, and better educated directors with previous board experience working at large and profitable firms tend to have more board positions. Moreover, directors working at companies in industries that tend to have smaller boards have fewer board positions. Next, directors working in countries with larger capital markets and belonging in the largest network components have more board positions. Lastly, the number of board positions directors have is not related to being located in countries switching from a non-binding gender target to a binding gender quota.

## Additional tests

### Quasi-experimental analyses

In order to get us closer to make causal inferences, we conduct a series of tests using a quasi-experimental design by applying two matching methods [71]. Specifically, we apply entropy balanced matching and coarsened exact matching (CEM), two matching methods for addressing causality concerns in observational data [72, 73]. We choose to apply both matching methods for robustness reasons as each method has its strengths and weaknesses. On the one hand, entropy balanced matching has an advantage over other matching procedures, like CEM, because it reduces the researcher’s discretion by finding globally optimal weights for achieving nearly identical distributions across underlying variables. That is, entropy balanced matching achieves near perfect covariate balance between treated and control samples [72, 74]. On the other hand, CEM is more widely used in social science research including research in corporate boards [75], and social networks [71].

#### Entropy balanced matching

The entropy balanced matching procedure is carried out in several steps. First, we match directors subject to affirmative action programs (treated sample) to other directors who share similar characteristics and have not been subject to any affirmative action programs (control sample). Because different affirmative action programs were passed in different countries in different years, we carry out our matching for treated directors in each country at a time. For example, we match directors in France -where a binding gender quota was passed in 2011- to directors in Bulgaria, Croatia, Cyprus, Czech Republic, Greece, Hungary, Liechtenstein, Lithuania, Malta, Monaco, Romania, Russia, Slovenia, Switzerland, Turkey, and Ukraine -where neither binding gender quotas nor non-binding gender targets have been passed. The characteristics we use to match directors are: age, education, board experience, maximum firm size, maximum firm profitability, large component, small board size sector, and country’s stock market size (%). Second, we use ordinary least squares estimations to compare the number of board positions directors hold between our treated and control samples, both before and after the passage of affirmative action programs. We refer to these settings as pre-quota/target and post-quota/target. When estimating the ordinary least squares, we include an interaction term between eigenvector centrality, gender, and a dummy variable for the treated sample. A significant interaction term in the period after the implementation of the affirmative action program is an indication of the possible causal effect affirmative action programs have -through networks- on the number of board positions directors hold (see S8 Table in S6 Appendix for details).

**Fig 5:**
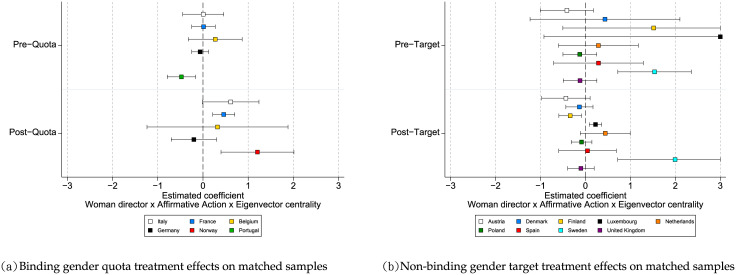
Entropy balanced matching results. In general, when comparing the pre and post implementation of affirmative action programs, the passage of binding gender quotas is associated with an increase in the network benefits of women directors while the passage of non-binding gender targets is not.

Fig 5 presents the coefficients and the 95% confidence intervals of the interaction term, *Woman director × Treated Sample × Eigenvector centrality*, estimated through ordinary least squares regressions on the matched samples for binding gender quotas (Fig 5a) and non-binding gender targets (Fig 5b). The results obtained from entropy balanced matching are in line with our main findings reported in Table 2. Considering the results in Fig 5a, two things stand out. First, in the pre-quota setting, the gender differences in network benefits do not vary between the treated and control samples. This is the case in four out of five countries (Italy, France, Belgium, and Germany), with a 95% confidence level. Second, in the post-quota setting, women in the treated samples tend to benefit more from their networks relative to the control samples. With a 95% confidence interval, women directors in three out of the five countries (Italy, France, and Norway) experience such benefits. When comparing both settings (pre- and post-quota settings), for most countries we observe a statistically significant increase in the network benefits women directors reap as a result of binding gender quotas (see Panel A in S8 Table in S6 Appendix). In contrast, Fig 5b shows that both in the pre and post-target settings, the gender differences in network benefits do not vary between treated and control samples. In general, women directors do not experience a statistically significant change in network benefits following the passage of non-binding gender targets (see Panel B in S8 Table in S6 Appendix). All in all, the results in Fig 5 suggest that the treatment effect for binding-gender quotas is significant for the increase in the network benefits women directors extract, whereas the treatment effect for non-binding gender targets is not. These analyses using entropy balanced matching further confirm our main findings.

#### CEM analysis

The CEM procedure is carried out in several steps. First, we match directors subject to affirmative action programs (treated sample) to other directors who share similar characteristics and have not been subject to any affirmative action programs (control sample). Because different affirmative action programs were implemented in different countries in different years, we carry out our matching for treated directors in each country at a time. For example, we match directors in France -where a binding gender quota was implemented in 2011- to directors in Bulgaria, Croatia, Cyprus, Czech Republic, Greece, Hungary, Liechtenstein, Lithuania, Malta, Monaco, Romania, Russia, Slovenia, Switzerland, Turkey, and Ukraine- where neither binding gender quotas nor non-binding gender targets have been implemented. The characteristics we use to match directors are: age, education, board experience, maximum firm size, maximum firm profitability, and large component. We coarsen all continuous variables by quartiles to control for the amount of imbalance in the matching solution. Second, we use ordinary least squares estimations to compare the number of board appointments between our treated and control samples both before and after the implementation of affirmative action programs. When estimating the ordinary least squares, we include an interaction term between eigenvector centrality, gender, and a dummy variable equal to one for the treated sample (directors subject to affirmative action programs), as well as the same control variables used in the main analysis. A significant interaction term in the period after the implementation of the affirmative action program is an indication of the possible causal effect affirmative action programs have -through networks- on the number of board positions directors hold (see S11 Table in S7 Appendix for details).

**Fig 6:**
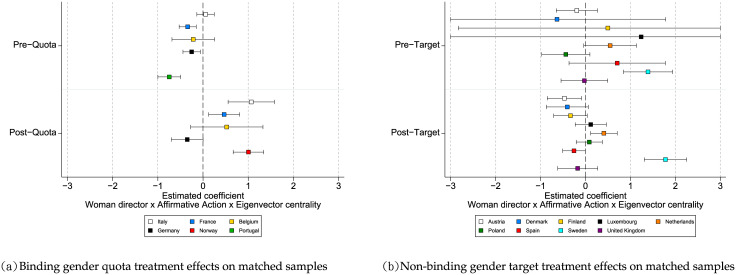
CEM results. In general, when comparing the pre and post implementation of affirmative action programs, the passage binding gender quotas is associated with an increase in the network benefits of women directors while the passage of non-binding gender targets is not.

Fig 6 presents the coefficients of the interaction term, *Woman director × Treated Sample × Eigenvector centrality*, estimated through ordinary least squares regressions on the matched samples for binding gender quotas (Fig 6a) and non-binding gender targets (Fig 6b). When comparing both the pre-quota and the post-quota settings in Fig 6a, for most countries we observe a statistically significant increase in the network benefits women directors reap as a result of gender binding quotas (see also S11 Table in S7 Appendix). In contrast, Fig 6b shows that both in the pre and post-target settings, the gender differences in network benefits do not vary between treated and control samples (see also S11 Table in S7 Appendix). In general, the results in Fig 6 suggest that the treatment effect for binding gender quotas is significant for the increase in the network benefits women directors extract, whereas the treatment effect for non-binding gender targets is not. These results are in line with our main findings in Table 2 and with the findings from the entropy balanced matching.

### Different estimation approaches

Next to quasi-experimental analyses, we carry out a series of robustness tests using different estimation approaches. In order to account for any time trends and country-specific characteristics related to the number of board positions directors obtain, we estimate our main analysis using a random effects panel data model with year and country fixed effects. In addition, since our dependent variable is a count measure we estimate our main analysis using a Poisson panel regression without and with year and country fixed effects. Table 3 summarizes the findings. All in all, we estimate ten different models: five for binding gender quotas (regressions (1) through (5)) and five for non-binding gender targets (regressions (6) through (10)). Of interest is the coefficient for the triple interaction *Woman director × Affirmative action program × Eigenvector centrality*. We find a positive relationship between the benefits women directors reap from their networks and the number of positions directors hold once binding gender quotas are passed as the positive and significant coefficients in regressions (1) through (5) show. In addition, we do not find a statistically significant relationship between the network benefits women directors reap from their networks and the number of positions directors hold once non-binding gender targets are passed (see regressions (6) through (10)). Overall, these results are consistent with our main results in Table 2.

**Table 3:** Robustness tests using different estimation approaches. Robustness tests using different estimation approaches for the relationship between affirmative action program, director’s gender, network position, and number of board positions show results that are consistent with the results from our main analysis. All regressions have the same set of control variables used in the main analysis.

	Binding gender quotas					Non-binding gender targets				
	(1)	(2)	(3)	(4)	(5)	(6)	(7)	(8)	(9)	(10)
	Random Effects with year FE	Random Effects with year and country FE	Panel Poisson	Panel Poisson with year FE	Panel Poisson with year and country FE	Random Effects with year FE	Random Effects with year and country FE	Panel Poisson	Panel Poisson with year FE	Panel Poisson with year and country FE
Woman director × Affirmative action program × Eigenvector centrality	0.016***	0.017***	0.009***	0.009***	0.010***	0.006	0.005	0.001	0.001	0.000
	[0.006]	[0.006]	[0.003]	[0.003]	[0.003]	[0.004]	[0.004]	[0.002]	[0.002]	[0.002]
Woman director × Affirmative action program	0.145***	0.129***	0.073***	0.065***	0.056**	-0.027	-0.012	-0.005	-0.011	0.007
	[0.028]	[0.029]	[0.023]	[0.023]	[0.022]	[0.021]	[0.021]	[0.021]	[0.021]	[0.021]
Affirmative action program × Eigenvector centrality	-0.007*	-0.007*	-0.005**	-0.004**	-0.004**	-0.006***	-0.007***	-0.002**	-0.002**	-0.003***
	[0.004]	[0.004]	[0.002]	[0.002]	[0.002]	[0.002]	[0.002]	[0.001]	[0.001]	[0.001]
Eigenvector centrality	0.024***	0.024***	0.014***	0.014***	0.014***	0.024***	0.024***	0.014***	0.014***	0.014***
	[0.002]	[0.002]	[0.001]	[0.001]	[0.001]	[0.002]	[0.002]	[0.001]	[0.001]	[0.001]
Affirmative action program	0.042***	-0.005	0.042***	0.067***	0.002	-0.008	0.003	-0.037***	-0.027***	-0.007
	[0.011]	[0.013]	[0.009]	[0.012]	[0.010]	[0.009]	[0.010]	[0.007]	[0.008]	[0.008]
Woman director	-0.089***	-0.106***	-0.047**	-0.038*	-0.042**	0.004	-0.035**	0.003	0.011	-0.018
	[0.021]	[0.021]	[0.019]	[0.019]	[0.019]	[0.014]	[0.014]	[0.012]	[0.013]	[0.013]
Year fixed effects	Yes	Yes	No	Yes	Yes	Yes	Yes	Yes	Yes	Yes
Country fixed effects	No	Yes	No	No	Yes	No	Yes	No	No	Yes
Observations	74289	74289	74289	74289	74289	88402	88402	88402	88402	88402
R-sq (within)	0.173	0.176				0.176	0.179			
R-sq (between)	0.200	0.209				0.181	0.197			
Log-likelihood			-88826.623	-88804.900	-88718.390			-105440.927	-105423.722	-105267.832

### Time-lagged analysis

**Fig 7:**
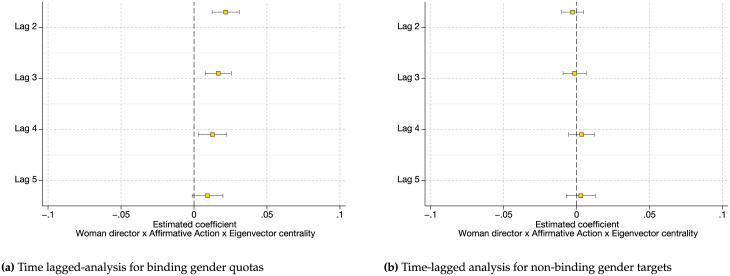
Time-lagged analysis for eigenvector centrality. Using different lags for the variable eigenvector centrality shows that the relationship between affirmative action program, gender and networks is consistent with our main findings. Margin plots are estimated from re-estimating our main regression using lagged eigenvector centrality measures two to five years prior. Figures include 95% confidence intervals.

In order to take into account any longer-lasting network effects beyond those captured in our main analysis, we carry out a series of time-lagged analyses. Specifically, similar to [66], we rerun our main analysis with eigenvector centrality measures lagged two to five years prior (as opposed to one year before). Fig 7 plots the estimated coefficients for the interaction term *Woman director × Affirmative action program × Eigenvector centrality* using different lags for the variable eigenvector centrality. We find that using different lags of eigenvector centrality shows the same patterns as those shown in our main analysis: the estimated coefficient for the interaction term in the binding gender quota setting is positive and significant while in the non-binding gender target setting it is not significant. This replication further validates the robustness and the temporal order of the relationships shown in our main analysis.

### Golden skirts and power inequality?

Although this paper finds that women and men directors reap more similar network benefits after the passage of binding gender quotas, we consider the possibility that quotas may in fact create inequalities among directors. In particular, we consider two types of inequalities associated with the number of board positions and social capital. First, we consider the possibility of a small set of women directors accumulating more board seats as a result of the passage of binding gender quotas. That is, we consider the possible emergence of *golden skirts* –a phenomenon documented in Norway, e.g. [27, 76], albeit diminishing in recent years [77]. Second, we consider the possibility of social capital becoming more concentrated among a select few directors, in particular among a few women directors, as a result of the passage of binding gender quotas. That is, we consider the possible emergence of power inequality among directors.

According to the golden skirts hypothesis, most women directors are expected to hold many directorships following the passage of binding quotas [27, pp.51]. Just like in [27], for this analysis, we compare the proportion of women directors, with respect to all directors, across the number of board positions they hold before and after the passage of quotas. S13 Table in S8 Appendix summarizes the findings. We note two things. First, we observe an increase in the proportion of directors who are women after the passage of quotas (from 5.61% to 22.39%). Second, we observe a slight disproportional overrepresentation of women directors holding two or more board positions (25.19% vs. 22.39%), three or more board positions (27.23% vs. 22.39%), four or more board positions (25.26% vs. 22.39%), and six or more board positions (22.64 vs. 22.39%) –after the passage of quotas–; however, women directors are slightly underrepresented among directors with five or more board positions (19.21% vs. 22.39%), and fully underrepresented among directors with seven or more board positions (0.00% vs. 22.39%). Hence, we do not observe a strong overrepresentation of women directors holding many board positions, especially when considering five or more board positions. Similar patterns emerge when we look at the count of unique prominent women directors (see Panel B in S14 Table in S8 Appendix). While the proportion of unique prominent women directors has increased after the passage of quotas from 6.25% to 26.03%, this change is rather proportional to the change in representation of unique women directors in general (6.97% before quotas and 22.53% after quotas). Thus, we do not observe a strong overrepresentation of unique prominent women directors after quotas (26.03% vs. 22.53%). Our findings suggest the golden skirts phenomenon in Europe is not as strong as the one previously documented in Norway by [27], where a disproportional representation of women directors holding multiple board positions has been documented after the passage of the quota; for example, 61.4% of directors holding three or more directorships were women, while 39.1% of all directors were women. In the case of our sample, rather than observing only a small set of women directors holding most board positions, we also see a larger group of women acting as directors on boards.

In line with the view that quotas may cause inequality among directors by concentrating their power, the social capital of directors is expected to become more concentrated among a select few directors and become even more concentrated among a few women directors after the passage of quotas [27, pp.51]. Just like in [27], for this analysis, we compare the standard deviation of our social capital measure for prominent directors (those with more than one board position) before and after the passage of quota, and the level of our social capital measure between prominent women and men directors after the passage of quotas. S14 Table in S8 Appendix summarizes the findings. We find that the standard deviation of eigenvector centrality, among prominent directors, decreases after the passage of quotas. Moreover, we find that the average eigenvector centrality of prominent women directors is lower than that of prominent men directors after the passage of quotas. These findings are in contrast with [27], who find an increase in the standard deviation of the betweenness centrality among Norwegian directors with women directors having higher levels of social capital than men directors after the passage of quotas, and associate these changes with the emergence of power inequality in favor of women directors. Therefore, our findings suggest that power inequality among directors has not increased after the passage of binding quotas.

Last, quotas critics argue that an easy workaround to be quota compliant is to select from the same pool of women directors that already have experience in public boards, instead of having a larger pool, resulting in few women holding many positions and skewing the equality debate behind quotas. To address this issue, we shift our focus to the count of unique woman directors holding positions in our sample (see S14 Table in S8 Appendix). We find that there has been an increase in the number of unique women directors. Interestingly, the average number of board positions prominent women directors hold does not increase after the passage of quotas suggesting that quotas are not likely to be associated with a concentration of board positions among a few prominent women directors.

All in all, we do not find strong evidence for the golden skirts phenomenon and no evidence for the power inequality phenomenon in our study. Instead, our main findings reported in Table 2 suggest that women directors can benefit more from their networks, after the passage of binding gender than before, bringing them more at par with the benefits men directors reap from their networks. In this way, our research provides evidence that binding gender quotas make corporate director networks a salient tool for hiring women and may help in leveling the playing field in the way these networks are used for achieving top management positions.

## Discussion and conclusion

Networks, or “who you know,” matter greatly for career advancement in general, and the number of board positions directors hold, in particular. Women are often presented as disadvantaged individuals not possessing the right social capital or not benefiting from their social capital enough to join top managerial positions on equal footing with men, leading to their underrepresentation in boards. The recent passage of affirmative action programs by European governments, however, raises the question of whether and to what extent network benefits for both men and women directors remain unchanged after the passage of these programs. The passage of affirmative action programs in Europe allows us to exploit the statistical properties of a quasi-natural experiment to study their moderating effect on the role of networks, and gender when explaining the number of board positions directors hold. Using multi-level regressions, we find that the presence of binding gender quotas enables women directors to derive more benefits from their networks. These results are further supported by entropy balanced matching analyses, CEM, time-lagged analyses, and different estimation approaches.

Our findings both support and enhance existing network theories in career advancement. Research has shown that crucial to career advancement is building relations with others who have the potential to help career-wise [12]. Indeed, we find that directors who have relations with more important individuals—holding board positions at institutions such as clubs, military, charitable, government, sporting, educational, and medical as well as in public and private companies worldwide– are better able to obtain board positions than directors who have relations with less important individuals. Moreover, in line with the research within the field of networks in career advancement that specifically considers the role of gender (e.g., [4, 10–13]), we find that, on average, women directors extract less network benefits than men before the passage of binding gender quotas. Reasons behind this lower extraction of network benefits are threefold. First, the “who-you-know” approach of recruiting board members tends to favors the selection of men to board positions due to in-group/out-group biases. Thus, women are more likely to be excluded as candidates even when having the right qualifications and connections within the network. Examples of these in-group/out-group biases include women needing to provide evidence of their legitimacy before fully leveraging their networks [4, 10]; women being excluded from job-relevant information flows in networks [11]; and women being unable to infiltrate influential circles [12]. Second, women may internalize discrimination and not pursue career advancement, such as board positions, to the same degree as men [78]. This internalization may happen even when the social capital stemming from networks that women have is the same as the social capital of men. Third, women tend to be less comfortable leveraging their networks than men [13]. That is to say, women, due to moral principles, are less inclined to use their networks as instruments to gain professional benefits. To illustrate, an interviewee –a woman in top management– indicated that “[she did not] really like the benefits definition of networks… It’s the exploitation aspect [she did not] like” preferring instead a relationship-based, communal approach to networks [13]. Altogether, selection committee members searching for candidates in their networks come across more often with men candidates having the right connections, qualifications, and in-group characteristics. This hiring practice, thus tends to exclude candidates not having the *correct* in-group characteristics in terms of gender, but who may be comparable to those chosen in terms of social capital and qualifications.

We enhance network theories in career advancement by showing that changes in context, in our case a change in the legal environment, matter for the benefits networks offer. There are two potential mechanisms that explain the increase in the network benefits women directors experience after the passage of quotas. The first mechanism concerns women’s inclusion to relevant corporate networks. Once binding gender quotas are passed, selection committee members more actively look for women in their networks; for example, by reaching both previously neglected areas of their networks and previously neglected candidates [79]. Through this more active search, recruiting committees are able to reach potential women candidates at higher rates than before [80]. As a result, women become more visible in the network of recruiting committees, and this visibility becomes more comparable to that of men. As the visibility of out-group members, in the recruitment process for board directors, becomes more equal to that of in-group members, women cease to be seen as the “other” and the influence of in-group members weakens [33], i.e. in-group/out-group biases lessen. With lower in-group/out-group biases, the ways networks are used to recruit directors become more inclusive. The second mechanism concerns women’s use of their networks for career advancement. With network benefits generally being lower for out-group members than for in-group members [10], women may under-utilize their networks for career advancement in anticipation of such lower benefits. That is, it is possible that women negatively self-select themselves by not actively using their networks as instruments for career advancement because they believe (whether it is true or not) that they have a low chance of getting board positions. However, women may change their perceptions about their likelihood of getting board positions because the passage of binding gender quotas signal to potential women candidates that they are welcome and encouraged to bring themselves forward as candidates [79]. As a result, women may feel more confident in using their networks making themselves more visible in the network by letting selection committee members know their interests and qualifications. Either mechanism results in women achieving a higher network visibility and allows them to derive more network benefits after the passage of binding gender quotas than before. However, considering that the recruitment process for board directors works predominantly through nominations by standing directors and CEOs [16], who tend to rely on networks to hire directors, we expect the first mechanism as having more influence after the passage of quotas. Our findings serve as an early indication that women’s improved network salience due to the passage of binding gender quotas can partially counterbalance the reasons behind their lower extraction of network benefits documented in the literature.

Our study also contributes to the research addressing the efficiency of affirmative action programs, for example [81], by taking into account the role of networks in the board recruitment process. We add to this discourse by showing that director networks play a role in the effectiveness of diversity programs. While social capital becomes more useful to women when binding gender quotas are passed, making the board selection process more comparable to the one for men, there is no change in the role of social capital under non-binding gender targets. The increase in the benefits of social capital under binding gender quotas, for women directors, may help in leveling the playing field across genders in achieving top management positions. From a policy standpoint, binding gender quotas appear to fuel the power of women’s networks counterbalancing the long-lasting dominance of the old-boys’ networks in board positions. In this regard, binding gender quotas function more efficiently as a policy instrument, in comparison to non-binding gender targets.

Our study suggests a number of directions for future research. The first area stems directly from the limitations of this study. Estimating causal effects without experimental data is as difficult as it is ingrained in social science research [82]. Although the matching methods and the battery of robustness tests we used in this research gets us closer toward being able to make causal inferences, we cannot exclude the possibility of other unobservable factors causing the gender differences in the number of board positions directors hold. One way to address this empirical limitation is to conduct an in-depth qualitative study where both men and women directors in countries with binding gender quotas and with non-binding gender targets are interviewed about the changes that the passage of affirmative action programs has had on their network benefits. Despite this limitation, we believe our results are at least suggestive that binding gender quotas in Europe change the role networks have had in perpetuating gender disparities in board representation.

Another area of further research concerns the long-term network dynamics associated with affirmative action programs. We find that women directors having relations with important members in the network are better able to hold board positions when there are binding gender quotas making them more at par with men directors. Yet, the consequences of our findings ought to be studied further. We propose two potential hypotheses about long-term networks dynamics for women directors obtaining board positions as a result of binding gender quotas. On the one hand, these directors may support other women to enhance their chances of career advancement thereby increasing women representation in corporate decision making. On the other hand, these directors may maximize their own professional gains without actively influencing women representation in corporate decision making. These long-term network dynamics may, in turn, depend on other contextual factors also worth investigating.

Another area of future research concerns other potential spillovers networks may have on the effectiveness of affirmative action programs beyond their formal fulfillment. For example, does a higher reliance on networks to hire directors after the passage of binding gender quotas help closing the gender wage gap? On the one hand, the literature on networks and managerial compensation would suggest that women obtaining board positions through their networks receive higher salaries than women who do not. This may possibly lead to higher average wages for women when there are binding gender quotas thus narrowing the gender wage gap under these legal settings. On the other hand, the literature on tokenism and social closure would suggest that networks only serve as a tool to find women candidates to fill up board positions without necessarily improving their wages.

Feeling the pressure from state-mandated quotas, companies are increasing the number of women representation in corporate boards throughout the world. With a 44% women representation in boards, France –a country with a binding gender quota– is now leading the way in board gender diversity among European firms [83]. This development can be partially attributed to the recognition of women in the networks of corporate directors. In the Netherlands—a country with a non-binding gender target– policy makers are now in the process of passing a binding gender quota with Minister Ingrid van Engelshoven announcing “We write history. We are breaking the old boys network and taking a major step towards equality and diversity at the top of the business” [84].

## Supporting information

S1 Appendix Measures. [14, 31, 65, 85–88].
(PDF)

S2 Appendix Sample construction. (PDF)

S3 Appendix Descriptives. (PDF)

S4 Appendix Additional details behind the marginal effects in the manuscript corresponding to the results in Table 2. [89, 90].
(PDF)

S5 Appendix Regressions with three-level categorical variable for affirmative action program. (PDF)

S6 Appendix Additional details behind entropy balanced matching analysis. (PDF)

S7 Appendix Additional details behind CEM analysis. (PDF)

S8 Appendix Additional details behind the golden skirts and power inequality analyses. [27].
(PDF)

S1 Fig (EPS)

S2 Fig (EPS)

S3 Fig (EPS)
