# Supplementary material for: Affirmative action programs and network benefits in the number of board positions
Source: PLoS One. 2020 Aug 4;15(8):e0236721. doi: 10.1371/journal.pone.0236721 (PMC7402479; doi:10.1371/journal.pone.0236721)
Supplement: S1 Appendix — [14, 31, 65, 85–88]. (PDF) [file pone.0236721.s001.pdf]

S1 Appendix. **Measures.** The following appendix provides detailed definitions and properties for all the metrics used in our analyses.

S1.1 Network. We create our director networks by transforming a bipartite graph into a one-mode network. A bipartite graph is mathematically defined as $G(U, S, E)$, where U and S are two different sets of nodes such that $V(G) = U(G) \cup S(G)$ and $U(G) \cap S(G) = \varnothing$. For each tie between $uv \in E(G)$ either $u \in U(G), v \in S(G)$ or $v \in U(G), u \in S(G)$. We project bipartite graph $G(U, S, E)$ for the node set U with respect to node set S to construct a one mode network $G'(U, E')$ where $V(G) = U$ and $(u_i, u_j) \in E(G')$.

A hypothetical example of a bipartite network and its one-mode projection is shown in S1 Fig . In the bipartite network in S1 Fig , the ties are created through directors sitting at boards in clubs, military, charitable, government, sporting, educational, and medical institutions as well as in public and private companies worldwide. In the corresponding one-mode network projection in S1 Fig , the ties are created through directors sitting at the same corporate boards.

Fig S1. Hypothetical example of a bipartite network and its one-mode projection for directors in corporate boards.

S1.2 Eigenvector centrality. *Eigenvector centrality* in one-mode network $G'(U, E')$ is calculated as:

$$x = \frac{1}{\lambda} \sum_{j \in U(G')} a_{i,j} x_j \qquad (1)$$

where u is a node in one mode network $G'(U, E')$; $a_{i,j}$ represents the entries in the adjacency matrix $\mathbf{A}$, i.e. $a_{i,j} = 1$ when node u_i is tied to node u_j, and $a_{i,j} = 0$ otherwise; λ is a constant eigenvalue. The centrality of a node is proportional to the sum of the

centralities of the nodes to which it is connected [65]. Following common practice, we normalize eigenvector centrality to allow for longitudinal comparisons [85, 86], and lag this measure by one year in all analyses.

S1.3 Number of board positions. The variable *Number of board positions* is our dependent variable and it measures the number of European board positions each director in our sample has. Since our setting is the European market for directors, our dependent variable captures European board positions.

S1.4 Woman director. The variable *Woman director* is a dummy variable that is equal to one when the director is a woman and zero otherwise.

S1.5 Affirmative action program Because we study two types of affirmative action programs, we construct a dummy variable for each:

S1.5a Binding gender quota. The variable *Binding gender quota* is a country-level dummy variable that is equal to one when a director sits in boards in a country with a binding gender quota and zero otherwise. All directors in our sample sit in boards within the same country.

S1.5b Non-binding gender target. The variable *Non-binding gender target* is a country-level dummy variable that is equal to one when a director sits in boards in a country with a non-binding gender target and zero otherwise. All directors in our sample sit in boards within the same country.

S1.6 Controls. We control for a set of factors that can influence the number of board positions directors have. These factors account for director-level, firm-level, industry-level, country-level, and network-level characteristics. We lag all control variables by one year in all analyses. Our director-level characteristics include board experience, age, education (*Graduate degree*). We include the variable *Board experience* since a director's previous board experience is an important factor in their rate in joining subsequent boards [14]. This variable is equal to one if a director sat in at least two board positions and zero otherwise. Next, we account for a director's age through

the variables *Age* and Age^2. Since, directors obtain more board positions as they get older, we account for their age (in years). But, since the effect of age might not be linear, we also account for the higher-order effect age may have through the variable Age^2. We account for the directors' education background with the dummy variable *Graduate degree* which is equal to one if the director has a graduate degree (Master's degree or above) and zero otherwise.

Our firm-level characteristics include *Maximum firm size* and *Maximum firm profitability*. These variables account for important financial characteristics of the firms where our sample of directors sit [87]. *Maximum firm size* is measured as the maximum total assets of all the firms where they act as directors, and *Maximum firm profitability* is measured as the maximum return on assets of all the firms where they act as directors. We choose the maximum instead of the average values to capture the hiring strategy typically used when seeking individuals for board positions that focuses on hiring those reputable individuals from the largest firms [31].

Our industry-level characteristic is *Small board size sector*. With this variable we account for directors predominantly working in sectors characterized by smaller boards. Controlling for this allows us to tease out the possibility of the number of board seats directors hold being driven by the sector where they work. The threshold for classifying small board sizes corresponds to values below the median board size across sectors of 9.877, where sector board size ranges between a minimum of 4.026 and a maximum of 17.149. Interestingly, the sectors we identify as having small board sizes coincide with those sectors that tend to be men-dominated. In our sample, these sectors include Aerospace & Defence, Automobiles & Parts, Business Services, Chemicals, Construction & Building Materials, Containers & Packaging, Electricity, Electronic & Electrical Equipment, Engineering & Machinery, Forestry& Paper, Health, Information Technology Hardware, Investment Companies, Leisure & Hotels, Mining, Oil & Gas, Pharmaceuticals and Biotechnology, Private Equity, Publishing, Real Estate, Renewable Energy, Software & Computer Services, Speciality & Other Finance, Steel & Other Metals, Transport, and Utilities - Other. The remaining industries in our sample are Banks, Beverages, Blank Check / Shell Companies, Clothing & Personal Products, Consumer Services, Diversified Industrials, Education, Food & Drug Retailers, Food Producers & Processors, General Retailers, Household Products, Insurance, Legal,

Leisure Goods, Life Assurance, Media & Entertainment, Telecommunication Services, Tobacco, and Wholesale Trade. We measure *Small board size sector* as the concentration of board positions directors have in sectors identified as having small board sizes. As such, this variable ranges between zero and one.

Our country-level characteristics are *Country's stock market size (%)* and *Affirmative action program switch*. In order to control for the possible supply-driven effects in the number of board seats directors can attain, we control for the size of a country's stock market relative to the size of the economy. We focus on the stock market because our study concerns the board seat positions directors obtain in stock-listed companies. This variable is measured as the ratio between the value of listed shares (stock market capitalization) and gross domestic product (GDP) times 100, and it is obtained from the World Bank [88]. Since data for all years is not always available, we average this ratio across each country. As there is not much yearly variation within countries, using an average deems a suitable aggregation approach. Since three countries in our sample switch from having a non-binding gender target to a binding gender quota, see Table 1, we also include the variable *Affirmative action program switch* which is equal to one when the switch happens in each of the three countries.

Lastly, our network-level characteristic is the dummy variable *Large component*. We include this variable in our analysis to control for the possible variation in director's network outreach and hence the number of positions that can be obtained. We begin by identifying network components within the director networks by detecting maximally connected sub-graphs where all nodes are connected with each other through a path. We find that about 90% of directors belong to the two largest components and therefore we set the dummy variable *Large component* equal to one when directors belong to either of the largest components and zero otherwise.
