# Supplementary material for: Affirmative action programs and network benefits in the number of board positions
Source: PLoS One. 2020 Aug 4;15(8):e0236721. doi: 10.1371/journal.pone.0236721 (PMC7402479; doi:10.1371/journal.pone.0236721)
Supplement: S2 Appendix — (PDF) [file pone.0236721.s002.pdf]

S2 Appendix. Sample construction

S1 Table presents the data sources used for constructing our variables. As indicated in the manuscript, our data mainly comes from BoardEx and Thomson Reuters Datastream accessible with licensing. We use the BoardEx core reports, version 2018, delivered in Excel files. For each of the variables derived from the BoardEx database, we provide the names of the core reports we extract the data from. We use the BoardEx

core reports, version 2018, delivered as Excel files directly from BoardEx. Researchers interested in obtaining licensing rights for using BoardEx can do so by e-mail at sales@boardex.com. Also, a demo can be requested at https://corp.boardex.com/request-relationship-mapping/. For each of the variables derived from the Thomson Reuters Datastream database, we provide the corresponding variable codes. Researchers interested in obtaining licensing rights for using Thomson Reuters Datastream can do so at https://www.refinitiv.com/en/contact-sales.

S1 Table. Measures and their data sources

Measure	Source
Board positions	Europe Director Profile (BoardEx)<br>Europe Senior Managers and Disclosed Earners Profile (BoardEx)<br>UK Director Profile (BoardEx)<br>UK Senior Managers and Disclosed Earners Profile (BoardEx)
Network<br>(eigenvector centrality<br>and large component)	Europe Director Network (BoardEx)<br>Europe Senior Managers and Disclosed Earners Network (BoardEx)<br>UK Director Network (BoardEx)<br>UK Senior Managers and Disclosed Earners Network (BoardEx)
Woman director	Europe Director Profile (BoardEx)<br>Europe Senior Managers and Disclosed Earners Profile (BoardEx)<br>UK Director Profile (BoardEx)<br>UK Senior Managers and Disclosed Earners Profile (BoardEx)
Board experience	Europe Director Profile (BoardEx)<br>EuropeSenior Managers and Disclosed Earners Profile (BoardEx)<br>UK Director Profile (BoardEx)<br>UK Senior Managers and Disclosed Earners Profile (BoardEx)
Age	Europe Director Profile (BoardEx)<br>Europe Senior Managers and Disclosed Earners Profile (BoardEx)<br>UK Director Profile (BoardEx)<br>UK Senior Managers and Disclosed Earners Profile (BoardEx)
Graduate degree	Europe Director Profile (BoardEx)<br>Europe Senior Managers and Disclosed Earners Profile (BoardEx)<br>UK Director Profile (BoardEx)<br>UK Senior Managers and Disclosed Earners Profile (BoardEx)
Maximum firm size	WC02999 (Thomson Reuters Datastream)
Maximum firm profitability	WC08326 (Thomson Reuters Datastream)
Small board size sector	Europe Director Profile (BoardEx)<br>Europe Senior Managers and Disclosed Earners Profile (BoardEx)<br>Europe Company Details (BoardEx)<br>UK Director Profile (BoardEx)<br>UK Senior Managers and Disclosed Earners Profile (BoardEx)<br>UK Company Details (BoardEx)
Country's stock market size	CM.MKT.I.LCAP.GD.ZS (World Bank)

Since our analysis uses director-level and firm-level characteristics, we exclude observations with missing director-level and firm-level characteristics. Our data set, before the exclusion of missing director-level and firm-level characteristics, contains 271,032 director-year observations of directors sitting in boards between 2000 and 2017. When considering director-level characteristics, we exclude observations without data

about gender (45), network (44,975), age (75,297), or graduate degree (27,048). Of the remaining 123,667 observations, we exclude those with missing firm-level characteristics, firm size (1,451) or firm performance (1,970). The final sample contains the remaining 120,246 director-year observations.

S2 Table compares the characteristics between the observations that enter our analysis with the observations that are excluded from our analysis due to missing director-level and firm-level characteristics. The excluded observations correspond to directors, who on average, hold fewer board positions in public firms (0.26 fewer boards), are less central in the network (0.19 smaller eigenvector centrality), are less than a year younger, and sit in smaller and less profitable firms. Additionally, 14% of the excluded observations are women directors as opposed to 12% in the sample observations; 7% of the excluded observations have board experience as opposed to 16% in the sample observations; and 53% of the excluded observations have a graduate education as opposed to 55% in the sample observations. While the differences between the sample observations and the excluded observations are statistically significant as indicated by the column *Coefficient Difference* in S2 Table , the differences we find are not surprising because it is likely that there is less information available for younger directors with less experience, working at smaller firms.

We note that our excluded observations, due to missing data, are not substantially biased toward women directors. While 12% of our sample of observations are women directors, 14% of the excluded observations are women directors. This indicates that women are slightly more likely to have missing data; however, the difference while statistically significant is equal to 2%. Furthermore, with the exception of eigenvector centrality, there is a consistent direction in the average differences between the sample observations and the excluded observations among men (Panel B in S2 Table), and women directors (Panel C in S2 Table). The average eigenvector centrality is larger for women directors in the excluded observations with respect to the sample observations. This difference may suggest that in our analyses we capture the effects of affirmative action programs not only for the most central women in the network of directors holding board positions at institutions such as clubs, military, charitable, government, sporting, educational, and medical as well as in public and private companies worldwide, but also those that are less central.

S2 Table. Mean comparison for director-level and firm-level characteristics between observations in our sample and those excluded from our sample. The columns labeled *Coefficient Difference* report the difference in means between the sample of observations and the excluded observations and their corresponding significance levels. * $p < 0.10$, ** $p < 0.05$, *** $p < 0.010$

Panel A: All directors	Sample observations		Excluded observations		
	Average	N	Average	N	Coefficient Difference
Board positions	1.36	120246	1.10	150786	−0.26***
Woman director	0.12	120246	0.14	150741	0.02***
Eigenvector centrality	0.81	120246	0.61	105775	−0.19***
Board experience	0.16	120246	0.07	150786	−0.10***
Age	55.71	120246	54.83	58238	−0.88***
Graduate degree	0.55	120246	0.53	87814	−0.02***
Maximum firm size	15.03	120246	14.16	109261	−0.87***
Maximum firm profitability	0.04	120246	0.04	104920	−0.01***

Panel B: Men directors	Sample observations		Excluded observations		
	Average	N	Average	N	Coefficient Difference
Board positions	1.36	105894	1.11	130222	−0.25***
Eigenvector centrality	0.85	105894	0.61	92203	−0.25***
Board experience	0.16	105894	0.07	130222	−0.09***
Age	56.26	105894	55.47	49326	−0.79***
Graduate degree	0.54	105894	0.52	76910	−0.02***
Maximum firm size	15.01	105894	14.14	95832	−0.88***
Maximum firm profitability	0.04	105894	0.03	92030	−0.01***

Panel C: Women directors	Sample observations		Excluded observations		
	Average	N	Average	N	Coefficient Difference
Board positions	1.37	14352	1.07	20519	−0.30***
Eigenvector centrality	0.47	14352	0.68	13563	0.21***
Board experience	0.16	14352	0.05	20519	−0.11***
Age	51.66	14352	51.28	8909	−0.38**
Graduate degree	0.59	14352	0.55	10874	−0.05***
Maximum firm size	15.18	14352	14.34	13420	−0.84***
Maximum firm profitability	0.04	14352	0.04	12881	−0.01***
