# Supplementary material for: Affirmative action programs and network benefits in the number of board positions
Source: PLoS One. 2020 Aug 4;15(8):e0236721. doi: 10.1371/journal.pone.0236721 (PMC7402479; doi:10.1371/journal.pone.0236721)
Supplement: S3 Appendix — (PDF) [file pone.0236721.s003.pdf]

S3 Appendix. Descriptives

S3 Table. Descriptive statistics of overall data for three different samples: pooled, binding gender quota analysis (Model (1) in Table 2 in manuscript), non-binding gender target analysis (Model (2) in Table 2 in manuscript. The sample mean is presented in each of the columns of the table. The columns labeled *Coefficient Difference* report the difference in means between the observations under affirmative action programs and those without within each sample and their corresponding significance levels. * $p < 0.10$, ** $p < 0.05$, *** $p < 0.010$

	Pooled Sample	Binding gender quota sample			Non-binding gender target sample		
		Binding gender quota No	Binding gender quota Yes	Coefficient Difference	Non-binding gender target No	Non-binding gender target Yes	Coefficient Difference
Board positions	1.360	1.367	1.341	−0.026***	1.372	1.288	−0.084***
Eigenvector centrality	0.808	1.715	0.229	−1.485***	1.062	1.033	−0.029
Woman director	0.119	0.065	0.224	0.159***	0.104	0.084	−0.020***
Board experience	0.162	0.165	0.161	−0.003	0.164	0.143	−0.021***
Age	55.711	55.354	55.871	0.517***	55.706	55.945	0.238***
Graduate degree	0.546	0.529	0.535	0.006	0.548	0.549	0.002
Maximum firm size	15.034	15.345	14.534	−0.811***	15.084	15.283	0.199***
Maximum firm profitability	0.044	0.048	0.028	−0.020***	0.044	0.040	−0.004***
Large component	0.943	0.930	0.931	0.001	0.935	0.936	0.002
Small board size sector	0.602	0.595	0.693	0.098***	0.621	0.635	0.014***
Country's stock market size (%)	4.179	4.143	4.160	0.017***	4.259	4.070	−0.190***
Observations	120246	50516	23773	74289	63910	24492	88402

S5 Table shows yearly eigenvector centrality mean, and mean differences between women and men directors for our total sample of directors (Panel A), directors in countries with a binding gender quota (before and after)(Panel B), and directors in countries with a non-binding gender target (before and after)(Panel C). The yearly distribution of eigenvector centrality for the entire sample of men and women directors (Panel A) shows that while there are differences between the average eigenvector centrality for men and women directors, these differences are only statistically significant in six of the 18 years of our sample. In most years women directors have a lower eigenvector centrality than men directors. Important to note is that for the samples of men and women directors directors in countries with a binding gender quota (Panel B), and directors in countries with a non-binding gender target (Panel C) there are not any "after quota" observations before 2003 since the first binding gender quota was passed in 2003 in Norway. Similarly, the first non-binding gender target was passed in 2007 in Spain. Last, with Portugal being the last country passing a binding gender quota, there are no "before quota" observations in 2017.

When considering differences before and after the passage of binding gender quotas and non-binding gender targets (Panel B and Panel C in S5 Table), we note the following. Women directors, on average, have a higher eigenvector centrality than men directors before quotas and a lower eigenvector centrality after quotas. These differences are statistically significant at the 10% level. Before the passage of non-binding gender targets, the average eigenvector centrality for women directors is

S4 Table . Pairwise correlations

	1	2	3	4	5	6	7	8	9	10	11
1. Board positions	1.000										
2. Eigenvector centrality	0.218	1.000									
3. Woman director	0.005	−0.029	1.000								
4. Board experience	0.364	0.031	−0.003	1.000							
5. Age	0.081	0.020	−0.155	0.047	1.000						
6. Graduate degree	0.066	0.024	0.035	0.049	−0.032	1.000					
7. Maximum firm size	0.218	0.240	0.023	0.099	0.136	−0.027	1.000				
8. Maximum firm profitability	0.113	0.007	0.004	0.052	0.007	−0.016	0.162	1.000			
9. Large component	0.096	0.046	0.021	0.082	0.055	0.046	0.240	0.019	1.000		
10. Small board size sector	−0.228	−0.051	−0.020	−0.133	−0.017	0.020	−0.329	−0.073	−0.070	1.000	
11. Country’s stock market size (%)	0.036	−0.096	0.028	0.028	0.107	0.095	−0.099	0.013	0.050	0.031	1.000

S5 Table . Yearly eigenvector centrality mean, and mean differences between women and men directors for our total sample of directors, directors in countries with a binding gender quota (before and after), and directors in countries with a non-binding gender target (before and after).

	Panel A: Entire sample			Panel B: Binding gender quota sample						Panel C: Non-binding gender target sample					
				Before Quota			After Quota			Before Target			After Target		
	Women directors	Men directors	Coefficient Difference	Women directors	Men directors	Coefficient Difference	Women directors	Men directors	Coefficient Difference	Women directors	Men directors	Coefficient Difference	Women directors	Men directors	Coefficient Difference
2000	1.34	1.93	−0.598	2.60	3.46	−0.86				1.89	2.43	−0.54			
2001	0.91	1.52	−0.606*	1.70	2.59	−0.89				1.18	1.89	−0.71			
2002	0.85	1.53	−0.683**	1.61	2.57	−0.96				1.08	1.91	−0.83			
2003	1.09	1.79	−0.695*	2.77	3.36	−0.59	0.01	0.01	0.00	1.37	2.24	−0.87			
2004	1.12	1.66	−0.537*	2.32	2.78	−0.46	0.00	0.03	−0.03	1.62	2.19	−0.57			
2005	1.17	1.54	−0.366	2.26	2.47	−0.21	0.01	0.00	0.01***	1.84	2.02	−0.18			
2006	0.99	1.33	−0.333	2.10	2.15	−0.05	0.01	0.00	0.01**	1.62	1.75	−0.13			
2007	1.10	1.73	−0.629**	2.53	2.95	−0.42	0.01	0.00	0.01	2.19	2.58	−0.39	0.11	0.21	−0.10
2008	0.65	1.24	−0.598**	1.52	2.15	−0.63	0.00	0.00	0.00	1.79	2.54	−0.75	0.15	0.13	0.02
2009	1.51	1.62	−0.108	3.63	2.98	0.65	0.07	0.05	0.02	4.26	3.35	0.91	0.16	0.16	0.00
2010	1.60	1.46	0.137	3.71	2.64	1.07*	0.03	0.03	0.00	0.18	0.11	0.07	2.62	1.93	0.69*
2011	0.91	0.91	0.004	5.56	2.89	2.67***	0.11	0.16	−0.05	0.16	0.18	−0.02	2.83	1.72	1.10**
2012	0.77	0.65	0.119	4.63	2.09	2.54***	0.08	0.10	−0.02	0.11	0.10	0.01	2.50	1.23	1.27***
2013	0.68	0.56	0.117	3.53	1.87	1.66***	0.06	0.06	0.00	0.07	0.07	0.00	2.04	1.14	0.90**
2014	0.62	0.54	0.072	3.25	1.81	1.44***	0.05	0.04	0.01	0.06	0.05	0.01	1.91	1.09	0.82***
2015	0.55	0.50	0.052	0.01	0.01	0.00	0.76	0.91	−0.15	0.88	0.96	−0.08	0.07	0.09	−0.02
2016	0.05	0.11	−0.063	0.00	0.00	0.00	0.07	0.17	−0.10	0.08	0.17	−0.09	0.01	0.04	−0.03
2017	0.02	0.04	−0.026			0.02	0.06	−0.04		0.03	0.07	−0.04	0.01	0.04	−0.03
Total	0.47	0.85	−0.38***	2.92	2.55	0.38*	0.17	0.25	−0.08*	0.66	1.52	−0.86***	1.44	1.00	0.45***

lower than that of men directors, and the average eigenvector centrality for women directors is higher than that of men directors after the passage of non-binding gender targets. These differences are statistically significant at the 1% level. These results suggest that women directors are more likely to be in the "right" network before the passage of binding gender quotas and less likely to be in the "right" network after the passage of binding gender quotas. Yet, they benefit more from their networks after the passage of binding gender quotas. Whereas after the passage of non-binding gender targets, women directors are more likely to be in the "right" network but without reaping more benefits from them.
