# Supplementary material for: Affirmative action programs and network benefits in the number of board positions
Source: PLoS One. 2020 Aug 4;15(8):e0236721. doi: 10.1371/journal.pone.0236721 (PMC7402479; doi:10.1371/journal.pone.0236721)
Supplement: S4 Appendix — [89, 90]. (PDF) [file pone.0236721.s004.pdf]

S4 Appendix. Additional details behind the marginal effects in the manuscript corresponding to the results in Table 2.

In order to better understand the relationship between directors' networks, gender, affirmative action programs, and the number of board positions director hold, we compute and plot the marginal effects for each of these variables. All in all, we do this for each of the two models reported in Table 2. See S2 Fig and S3 Fig .

Fig S2. Marginal effects for (a) eigenvector centrality, (b) woman director, and (c) affirmative action program (binding gender quota) corresponding to Model (1) in Table 2 in the manuscript. All other variables are held constant. 95% confidence intervals included.

Fig S3. Marginal effects for (a) eigenvector centrality, (b) woman director, and (c) affirmative action program (non-binding gender target) corresponding to Model (2) in Table 2 in the manuscript. All other variables are held constant. 95% confidence intervals included.

For the binding gender quota model, we note the following. First, for both women and men directors, the marginal effects of eigenvector centrality for the number of board positions are positive and statistically significant before and after the passage of quotas. This result is in line with the general notion that networks are important in career advancement. The positive and statistically significant marginal effects, ranging between 0.019 and 0.037, are illustrated in S2(a) Fig . As eigenvector centrality is a continuous variable, the marginal effects are calculated taking the partial derivative with respect to eigenvector centrality. We then evaluate the partial derivative at the two values the interacting variables, women directors and binding gender quotas, take: zero or one. For women directors there is a larger increase in the number of board positions for each unit increase in eigenvector centrality after the passage of binding gender

quotas compared to before the passage of binding gender quotas. This is illustrated by a marginal effect equal to 0.035 (Woman director = 1, Binding gender quota = 1) vis-à-vis 0.019 (Woman director = 1, Binding gender quota = 0).

Second, for women directors –with respect to men directors– the change in the number of board positions is positive after the passage of binding gender quotas and negative before the passage of binding gender quotas. Irrespective of eigenvector centrality, women experience an increase in the number of boards they hold after the passage of binding gender quotas.This is illustrated by the positive and statistically significant marginal effects in S2(b) Fig ranging between 0.001 and 0.814 (Binding gender quota = 1, and eigenvector centrality = 0, 20, 40, 60, 80). We note, however, that the marginal effects of women directors at eigenvector centrality equal to zero are statistically significant only at a 90% confidence level. As woman director is a categorical variable, the marginal effects are calculated using the discrete change in the conditional mean of number of board positions as the variable woman director changes from zero to one, and then evaluating the interacting variables, eigenvector centrality and binding gender quota at different values while all other variables are held constant [89, 90].

Third, after the passage of binding gender quotas –with respect to before the passage of binding gender quotas– there is an increase in the number of board positions women directors hold irrespective of the value eigenvector centrality takes. This is illustrated in S2(c) Fig by the positive and statistically significant marginal effects ranging between 0.067 and 1.330 (Woman director = 1, and eigenvector centrality = 0, 20, 40, 60, 80).

For the non-binding gender target model, we note the following. First, for both women and men directors, the marginal effects of eigenvector centrality for the number of board positions are positive and statistically significant before and after the passage of non-binding gender targets. This result is in line with the general notion that networks are important in career advancement. The positive and statistically significant marginal effects, ranging between 0.015 and 0.039, are illustrated in S3(a) Fig . For women directors there is a smaller increase in the number of board positions for each unit increase in eigenvector centrality after the passage of non-binding gender targets in comparison to before the passage of non-binding gender targets. This is illustrated by a marginal effect equal to 0.015 (Woman director = 1, Non-binding gender target = 1)

vis-à-vis 0.025 (Woman director = 1, Non-binding gender target = 0).

Second, for women directors –with respect to men directors– the change in the number of board positions is negative both before and after the passage of non-binding gender targets. Overall, women directors hold fewer board positions than men directors irrespective of whether non-binding gender targets have been passed or not, and the value of eigenvector centrality. This is illustrated by the negative and statistically significant marginal effects in S3(b) Fig ranging between -1.024 and -0.007 (Non-binding gender target = 1, and eigenvector centrality = 0, 20, 40, 60, 80), and -1.146 and -0.028 (Non-binding gender target = 0, and eigenvector centrality = 0, 20, 40, 60, 80). Even though the change in the number of board positions women directors hold, compared to men, is less negative after the passage of non-binding gender targets, than before the passage of non-binding gender targets, this change is not statistically significant.

Third, the marginal effects for the passage of non-binding gender targets are negative for women directors irrespective of the values for eigenvector centrality. This is illustrated in S3(c) Fig by the negative and statistically significant marginal effects ranging between -0.0762 and 0.004 (Woman director = 1, and eigenvector centrality = 0, 20, 40, 60, 80).

In particular, S2(b), S2(c), S3(b), and S3(c) Figs show that the passage of binding gender quotas is associated with a positive and significant change in the number of board positions women directors hold while the passage of non-binding gender targets is not.
