# Supplementary material for: Affirmative action programs and network benefits in the number of board positions
Source: PLoS One. 2020 Aug 4;15(8):e0236721. doi: 10.1371/journal.pone.0236721 (PMC7402479; doi:10.1371/journal.pone.0236721)
Supplement: S5 Appendix — (PDF) [file pone.0236721.s005.pdf]

S5 Appendix. Regressions with three-level categorical variable for affirmative action program

S6 Table. Multi-level regression of number of board positions on director's gender, networks and affirmative action program types using a three-level categorical variable for affirmative action program. Regression has the same set of control variables used in the main analysis.

	Affirmative action program
	Quota and Target
Affirmative action program (Quota) × Woman director × Eigenvector centrality	0.026*** [0.006]
Affirmative action program (Target) × Woman director × Eigenvector centrality	0.003 [0.004]
Affirmative action program (Quota) × Woman director	0.071*** [0.016]
Affirmative action program (Target) × Woman director	0.054*** [0.020]
Affirmative action program (Quota) × Eigenvector centrality	-0.017*** [0.002]
Affirmative action program (Target) × Eigenvector centrality	-0.013*** [0.001]
Woman director × Eigenvector centrality	-0.016*** [0.003]
Affirmative action program (Quota)	0.015** [0.007]
Affirmative action program (Target)	-0.010 [0.007]
Eigenvector centrality	0.041*** [0.001]
Woman director	-0.062*** [0.013]
HLM estimated SD (constant)	0.105*** [0.016]
HLM estimated SD (residual)	0.649*** [0.001]
Observations	97975
LR Test	32881.343
Log-likelihood	-96728.470
