# Supplementary material for: Affirmative action programs and network benefits in the number of board positions
Source: PLoS One. 2020 Aug 4;15(8):e0236721. doi: 10.1371/journal.pone.0236721 (PMC7402479; doi:10.1371/journal.pone.0236721)
Supplement: S6 Appendix — (PDF) [file pone.0236721.s006.pdf]

S6 Appendix. Additional details behind entropy balanced matching analysis

Since our goal is to compare the network benefits before and after the passage of affirmative action programs, we split our sample into two groups one before the passage of affirmative action programs (pre) and the second one after the passage of affirmative action programs (post). For each of the two samples, we apply entropy balanced matching. S7 and S8 Tables present the means of our key variables for both the pre- and post- samples before and after matching for binding gender quotas and non-binding gender targets, respectively. Before matching, the means in the treated and control groups are mostly statistically significantly different (see *Before matching*

columns in S7 and S8 Tables). These differences indicate that the control group is not appropriate given the characteristics of the treated group. Thus, we apply entropy balanced matching to reduce the imbalance in our key variables. The application of the continuous weights determined by the entropy balanced matching process results in the means being exactly equal across the treated and control groups (see *After matching* columns in S7 and S8 Tables).

S7 Table . Descriptive statistics for pre- and post- binding gender quota analysis before and after entropy balanced matching.(a) Country: Norway

	Pre-Quota Before matching			Pre-Quota After matching		Post-Quota Before matching			Post-Quota After matching	
	Treated	Control	Coefficient Difference	Treated	Control	Treated	Control	Coefficient Difference	Treated	Control
Board experience	0.170	0.156	−0.014	0.170	0.170	0.181	0.156	−0.024***	0.181	0.181
Age	50.161	56.134	5.973***	50.161	50.164	52.183	56.134	3.951***	52.183	52.185
Graduate degree	0.574	0.593	0.018	0.574	0.575	0.619	0.593	−0.027**	0.619	0.619
Maximum firm size	13.132	15.512	2.380***	13.132	13.133	13.937	15.512	1.575***	13.937	13.938
Maximum firm profitability	−0.023	0.055	0.078***	−0.023	−0.023	0.040	0.055	0.015***	0.040	0.040
Large component	0.766	0.933	0.168***	0.766	0.766	0.937	0.933	−0.004	0.937	0.937
Small board size sector	0.732	0.547	−0.184***	0.732	0.731	0.650	0.547	−0.103***	0.650	0.650
Country's stock market size (%)	3.985	4.365	0.380***	3.985	3.986	3.985	4.365	0.380***	3.985	3.985
Observations	423	19065	19488	423	1005	4104	19065	23169	4104	17590

(b) Country: Italy

	Pre-Quota Before matching			Pre-Quota After matching		Post-Quota Before matching			Post-Quota After matching	
	Treated	Control	Coefficient Difference	Treated	Control	Treated	Control	Coefficient Difference	Treated	Control
Board experience	0.225	0.156	−0.069***	0.225	0.225	0.171	0.156	−0.015*	0.171	0.171
Age	57.394	56.134	−1.260***	57.394	57.394	57.919	56.134	−1.785***	57.919	57.919
Graduate degree	0.223	0.593	0.370***	0.223	0.223	0.281	0.593	0.311***	0.281	0.281
Maximum firm size	16.078	15.512	−0.566***	16.078	16.078	15.585	15.512	−0.073*	15.585	15.585
Maximum firm profitability	0.041	0.055	0.015***	0.041	0.041	0.031	0.055	0.024***	0.031	0.031
Large component	0.972	0.933	−0.038***	0.972	0.972	0.969	0.933	−0.035***	0.969	0.969
Small board size sector	0.385	0.547	0.162***	0.385	0.385	0.499	0.547	0.048***	0.499	0.499
Country's stock market size (%)	3.720	4.365	0.645***	3.720	3.720	3.720	4.365	0.645***	3.720	3.720
Observations	4079	19065	23144	4079	7460	4792	19065	23857	4792	11135

(c) Country: France

	Pre-Quota Before matching			Pre-Quota After matching		Post-Quota Before matching			Post-Quota After matching	
	Treated	Control	Coefficient Difference	Treated	Control	Treated	Control	Coefficient Difference	Treated	Control
Board experience	0.178	0.156	−0.022***	0.178	0.178	0.162	0.156	−0.006	0.162	0.162
Age	55.714	56.134	0.420**	55.714	55.714	57.156	56.134	−1.022***	57.156	57.155
Graduate degree	0.430	0.593	0.163***	0.430	0.430	0.478	0.593	0.115***	0.478	0.478
Maximum firm size	15.173	15.512	0.339***	15.173	15.173	14.577	15.512	0.935***	14.577	14.579
Maximum firm profitability	0.046	0.055	0.009***	0.046	0.046	0.018	0.055	0.037***	0.018	0.018
Large component	0.913	0.933	0.021***	0.913	0.913	0.925	0.933	0.008**	0.925	0.925
Small board size sector	0.638	0.547	−0.090***	0.638	0.638	0.701	0.547	−0.154***	0.701	0.701
Country's stock market size (%)	4.379	4.365	−0.014	4.379	4.379	4.379	4.365	−0.014	4.379	4.379
Observations	9149	19065	28214	9149	7460	11234	19065	30299	11234	11135

* $p < 0.10$, ** $p < 0.05$, *** $p < 0.010$

(d) Country: Belgium

	Pre-Quota Before matching			Pre-Quota After matching		Post-Quota Before matching			Post-Quota After matching	
	Treated	Control	Coefficient Difference	Treated	Control	Treated	Control	Coefficient Difference	Treated	Control
Board experience	0.193	0.156	−0.037***	0.193	0.193	0.180	0.156	−0.024**	0.180	0.180
Age	53.577	56.134	2.557***	53.577	53.577	56.458	56.134	−0.324	56.458	56.458
Graduate degree	0.643	0.593	−0.050***	0.643	0.643	0.676	0.593	−0.084***	0.676	0.676
Maximum firm size	14.593	15.512	0.919***	14.593	14.593	14.612	15.512	0.900***	14.612	14.614
Maximum firm profitability	0.059	0.055	−0.004	0.059	0.059	0.043	0.055	0.012***	0.043	0.043
Large component	0.937	0.933	−0.003	0.937	0.937	0.976	0.933	−0.043***	0.976	0.976
Small board size sector	0.592	0.547	−0.044***	0.592	0.592	0.649	0.547	−0.102***	0.649	0.649
Country's stock market size (%)	4.205	4.365	0.160***	4.205	4.205	4.205	4.365	0.160***	4.205	4.205
Observations	2835	19065	21900	2835	7460	2756	19065	21821	2756	11135

(e) Country: Germany

	Pre-Quota Before matching			Pre-Quota After matching		Post-Quota Before matching			Post-Quota After matching	
	Treated	Control	Coefficient Difference	Treated	Control	Treated	Control	Coefficient Difference	Treated	Control
Board experience	0.143	0.156	0.014***	0.143	0.143	0.137	0.156	0.019***	0.137	0.137
Age	53.982	56.134	2.152***	53.982	53.982	55.649	56.134	0.484**	55.649	55.650
Graduate degree	0.551	0.593	0.042***	0.551	0.551	0.519	0.593	0.074***	0.519	0.519
Maximum firm size	15.329	15.512	0.183***	15.329	15.329	14.833	15.512	0.679***	14.833	14.833
Maximum firm profitability	0.044	0.055	0.011***	0.044	0.044	0.032	0.055	0.023***	0.032	0.032
Large component	0.926	0.933	0.008**	0.926	0.926	0.915	0.933	0.019***	0.915	0.915
Small board size sector	0.676	0.547	−0.128***	0.676	0.676	0.732	0.547	−0.185***	0.732	0.732
Country's stock market size (%)	3.839	4.365	0.526***	3.839	3.839	3.839	4.365	0.526***	3.839	3.839
Observations	15746	19065	34811	15746	12994	5517	19065	24582	5517	5601

(f) Country: Portugal

	Pre-Quota Before matching			Pre-Quota After matching		Post-Quota Before matching			Post-Quota After matching	
	Treated	Control	Coefficient Difference	Treated	Control	Treated	Control	Coefficient Difference	Treated	Control
Board experience	0.149	0.156	0.007	0.149	0.149	0.099	0.156	0.058*	0.099	0.099
Age	54.925	56.134	1.209***	54.925	54.927	57.759	56.134	−1.625*	57.759	57.760
Graduate degree	0.469	0.593	0.124***	0.469	0.469	0.556	0.593	0.037	0.556	0.556
Maximum firm size	15.746	15.512	−0.234***	15.746	15.745	15.213	15.512	0.299	15.213	15.213
Maximum firm profitability	0.042	0.055	0.013***	0.042	0.042	0.031	0.055	0.025**	0.031	0.031
Large component	0.943	0.933	−0.010	0.943	0.943	0.951	0.933	−0.017	0.951	0.951
Small board size sector	0.424	0.547	0.123***	0.424	0.424	0.588	0.547	−0.041	0.588	0.588
Country's stock market size (%)	3.530	4.365	0.835***	3.530	3.531	3.530	4.365	0.835***	3.530	3.530
Observations	1926	19065	20991	1926	16751	162	19065	19227	162	1844

* $p < 0.10$, ** $p < 0.05$, *** $p < 0.010$

S8 Table . Descriptive statistics for pre- and post- non-binding gender target analysis before and after entropy balanced matching (a) Country: Sweden

	Pre-Target Before matching			Pre-Target After matching		Post-Target Before matching			Post-Target After matching	
	Treated	Control	Coefficient Difference	Treated	Control	Treated	Control	Coefficient Difference	Treated	Control
Board experience	0.194	0.156	−0.038***	0.194	0.194	0.193	0.156	−0.037***	0.193	0.193
Age	53.848	56.134	2.286***	53.848	53.848	56.276	56.134	−0.142	56.276	56.276
Graduate degree	0.677	0.593	−0.084***	0.677	0.677	0.691	0.593	−0.099***	0.691	0.691
Maximum firm size	13.276	15.512	2.236***	13.276	13.281	13.863	15.512	1.649***	13.863	13.863
Maximum firm profitability	0.046	0.055	0.009***	0.046	0.046	0.051	0.055	0.005*	0.051	0.051
Large component	0.966	0.933	−0.032***	0.966	0.966	0.991	0.933	−0.058***	0.991	0.991
Small board size sector	0.559	0.547	−0.011	0.559	0.558	0.593	0.547	−0.045***	0.593	0.593
Country's stock market size (%)	4.513	4.365	−0.148***	4.513	4.513	4.513	4.365	−0.148***	4.513	4.513
Observations	4667	19065	23732	4667	6318	4419	19065	23484	4419	12277

(b) Country: Finland

	Pre-Target Before matching			Pre-Target After matching		Post-Target Before matching			Post-Target After matching	
	Treated	Control	Coefficient Difference	Treated	Control	Treated	Control	Coefficient Difference	Treated	Control
Board experience	0.215	0.156	−0.058***	0.215	0.215	0.213	0.156	−0.057***	0.213	0.213
Age	55.458	56.134	0.676	55.458	55.458	56.313	56.134	−0.179	56.313	56.311
Graduate degree	0.861	0.593	−0.268***	0.861	0.861	0.861	0.593	−0.269***	0.861	0.861
Maximum firm size	14.864	15.512	0.648***	14.864	14.864	14.516	15.512	0.996***	14.516	14.515
Maximum firm profitability	0.085	0.055	−0.029***	0.085	0.085	0.059	0.055	−0.004	0.059	0.059
Large component	0.952	0.933	−0.018	0.952	0.952	0.996	0.933	−0.063***	0.996	0.995
Small board size sector	0.516	0.547	0.032	0.516	0.516	0.571	0.547	−0.024*	0.571	0.572
Country's stock market size (%)	4.766	4.365	−0.401***	4.766	4.766	4.766	4.365	−0.401***	4.766	4.765
Observations	624	19065	19689	624	4107	2150	19065	21215	2150	14488

(c) Country: Denmark

	Pre-Target Before matching			Pre-Target After matching		Post-Target Before matching			Post-Target After matching	
	Treated	Control	Coefficient Difference	Treated	Control	Treated	Control	Coefficient Difference	Treated	Control
Board experience	0.167	0.156	−0.011	0.167	0.167	0.131	0.156	0.025**	0.131	0.131
Age	54.526	56.134	1.608***	54.526	54.525	54.930	56.134	1.204***	54.930	54.929
Graduate degree	0.756	0.593	−0.163***	0.756	0.756	0.774	0.593	−0.182***	0.774	0.774
Maximum firm size	15.238	15.512	0.274***	15.238	15.237	14.989	15.512	0.523***	14.989	14.988
Maximum firm profitability	0.085	0.055	−0.029***	0.085	0.085	0.069	0.055	−0.014***	0.069	0.069
Large component	0.999	0.933	−0.066***	0.999	0.998	0.997	0.933	−0.063***	0.997	0.996
Small board size sector	0.523	0.547	0.024	0.523	0.523	0.553	0.547	−0.005	0.553	0.553
Country's stock market size (%)	4.023	4.365	0.342***	4.023	4.023	4.023	4.365	0.342***	4.023	4.023
Observations	1175	19065	20240	1175	6318	1796	19065	20861	1796	12277

(d) Country: Poland

	Pre-Target Before matching			Pre-Target After matching		Post-Target Before matching			Post-Target After matching	
	Treated	Control	Coefficient Difference	Treated	Control	Treated	Control	Coefficient Difference	Treated	Control
Board experience	0.088	0.156	0.068***	0.088	0.088	0.105	0.156	0.051***	0.105	0.105
Age	49.704	56.134	6.430***	49.704	49.704	51.660	56.134	4.474***	51.660	51.661
Graduate degree	0.584	0.593	0.009	0.584	0.584	0.634	0.593	−0.042**	0.634	0.634
Maximum firm size	15.630	15.512	−0.118	15.630	15.630	15.569	15.512	−0.057	15.569	15.569
Maximum firm profitability	0.067	0.055	−0.012*	0.067	0.067	0.042	0.055	0.014***	0.042	0.042
Large component	0.951	0.933	−0.017	0.951	0.951	0.980	0.933	−0.047***	0.980	0.980
Small board size sector	0.287	0.547	0.261***	0.287	0.287	0.376	0.547	0.171***	0.376	0.376
Country's stock market size (%)	3.499	4.365	0.866***	3.499	3.499	3.499	4.365	0.866***	3.499	3.499
Observations	466	19065	19531	466	6318	1365	19065	20430	1365	12277

(e) Country: Austria

	Pre-Target Before matching			Pre-Target After matching		Post-Target Before matching			Post-Target After matching	
	Treated	Control	Coefficient Difference	Treated	Control	Treated	Control	Coefficient Difference	Treated	Control
Board experience	0.191	0.156	−0.035*	0.191	0.191	0.132	0.156	0.024***	0.132	0.132
Age	53.990	56.134	2.144***	53.990	53.992	54.810	56.134	1.324***	54.810	54.813
Graduate degree	0.575	0.593	0.018	0.575	0.575	0.544	0.593	0.049***	0.544	0.544
Maximum firm size	16.003	15.512	−0.491***	16.003	16.003	15.551	15.512	−0.039	15.551	15.550
Maximum firm profitability	0.043	0.055	0.012**	0.043	0.043	0.043	0.055	0.013***	0.043	0.043
Large component	0.975	0.933	−0.042***	0.975	0.975	0.926	0.933	0.007	0.926	0.926
Small board size sector	0.558	0.547	−0.011	0.558	0.558	0.619	0.547	−0.072***	0.619	0.619
Country's stock market size (%)	3.362	4.365	1.003***	3.362	3.363	3.362	4.365	1.003***	3.362	3.364
Observations	602	19065	19667	602	4107	2983	19065	22048	2983	14488

(f) Country: Netherlands

	Pre-Target Before matching			Pre-Target After matching		Post-Target Before matching			Post-Target After matching	
	Treated	Control	Coefficient Difference	Treated	Control	Treated	Control	Coefficient Difference	Treated	Control
Board experience	0.171	0.156	−0.015	0.171	0.171	0.131	0.156	0.025***	0.131	0.131
Age	55.543	56.134	0.591**	55.543	55.541	57.020	56.134	−0.886***	57.020	57.017
Graduate degree	0.598	0.593	−0.005	0.598	0.598	0.629	0.593	−0.036***	0.629	0.628
Maximum firm size	14.800	15.512	0.712***	14.800	14.800	14.879	15.512	0.633***	14.879	14.883
Maximum firm profitability	0.041	0.055	0.014***	0.041	0.041	0.038	0.055	0.017***	0.038	0.038
Large component	0.975	0.933	−0.042***	0.975	0.974	0.959	0.933	−0.025***	0.959	0.958
Small board size sector	0.581	0.547	−0.034**	0.581	0.581	0.651	0.547	−0.104***	0.651	0.651
Country's stock market size (%)	4.480	4.365	−0.115***	4.480	4.480	4.480	4.365	−0.115***	4.480	4.479
Observations	2234	19065	21299	2234	4107	4122	19065	23187	4122	14488

(g) Country: Spain

	Pre-Target Before matching			Pre-Target After matching		Post-Target Before matching			Post-Target After matching	
	Treated	Control	Coefficient Difference	Treated	Control	Treated	Control	Coefficient Difference	Treated	Control
Board experience	0.227	0.156	−0.071***	0.227	0.227	0.178	0.156	−0.021***	0.178	0.178
Age	56.543	56.134	−0.409	56.543	56.542	58.619	56.134	−2.485***	58.619	58.619
Graduate degree	0.429	0.593	0.164***	0.429	0.429	0.490	0.593	0.103***	0.490	0.490
Maximum firm size	16.309	15.512	−0.797***	16.309	16.307	16.134	15.512	−0.622***	16.134	16.134
Maximum firm profitability	0.060	0.055	−0.005*	0.060	0.060	0.045	0.055	0.010***	0.045	0.045
Large component	0.972	0.933	−0.038***	0.972	0.971	0.958	0.933	−0.025***	0.958	0.958
Small board size sector	0.496	0.547	0.051***	0.496	0.496	0.574	0.547	−0.027***	0.574	0.574
Country's stock market size (%)	4.348	4.365	0.017	4.348	4.348	4.348	4.365	0.017	4.348	4.348
Observations	1835	19065	20900	1835	3136	5112	19065	24177	5112	15459

(h) Country: United Kingdom

	Pre-Target Before matching			Pre-Target After matching		Post-Target Before matching			Post-Target After matching	
	Treated	Control	Coefficient Difference	Treated	Control	Treated	Control	Coefficient Difference	Treated	Control
Board experience	0.080	0.156	0.077***	0.080	0.080	0.096	0.156	0.061***	0.096	0.096
Age	56.806	56.134	−0.672*	56.806	56.806	58.666	56.134	−2.532***	58.666	58.666
Graduate degree	0.503	0.593	0.089***	0.503	0.503	0.477	0.593	0.116***	0.477	0.477
Maximum firm size	13.644	15.512	1.868***	13.644	13.644	13.780	15.512	1.732***	13.780	13.779
Maximum firm profitability	0.021	0.055	0.034***	0.021	0.021	0.040	0.055	0.016***	0.040	0.040
Large component	0.945	0.933	−0.012	0.945	0.945	0.993	0.933	−0.060***	0.993	0.993
Small board size sector	0.636	0.547	−0.089***	0.636	0.636	0.678	0.547	−0.130***	0.678	0.678
Country's stock market size (%)	4.705	4.365	−0.340***	4.705	4.705	4.705	4.365	−0.340***	4.705	4.705
Observations	1570	19065	20635	1570	8676	1347	19065	20412	1347	9919

(i) Country: Luxembourg

	Pre-Target Before matching			Pre-Target After matching		Post-Target Before matching			Post-Target After matching	
	Treated	Control	Coefficient Difference	Treated	Control	Treated	Control	Coefficient Difference	Treated	Control
Board experience	0.141	0.156	0.015	0.141	0.142	0.078	0.156	0.078***	0.078	0.078
Age	53.319	56.134	2.815***	53.319	53.328	57.371	56.134	−1.237***	57.371	57.371
Graduate degree	0.607	0.593	−0.014	0.607	0.607	0.592	0.593	0.000	0.592	0.592
Maximum firm size	14.936	15.512	0.576***	14.936	14.939	14.486	15.512	1.026***	14.486	14.486
Maximum firm profitability	0.067	0.055	−0.012*	0.067	0.067	0.039	0.055	0.016***	0.039	0.039
Large component	0.861	0.933	0.073***	0.861	0.861	0.902	0.933	0.032***	0.902	0.902
Small board size sector	0.568	0.547	−0.021	0.568	0.567	0.674	0.547	−0.127***	0.674	0.674
Country's stock market size (%)	4.833	4.365	−0.469***	4.833	4.832	4.833	4.365	−0.469***	4.833	4.833
Observations	417	19065	19482	417	5208	1310	19065	20375	1310	13387

(j) Country: Iceland

	Pre-Target Before matching			Pre-Target After matching		Post-Target Before matching			Post-Target After matching	
	Treated	Control	Coefficient Difference	Treated	Control	Treated	Control	Coefficient Difference	Treated	Control
Board experience	0.146	0.156	0.011	0.146	0.146	0.062	0.156	0.094**	0.062	0.062
Age	52.748	56.134	3.386***	52.748	52.749	54.858	56.134	1.276	54.858	54.857
Graduate degree	0.523	0.593	0.069	0.523	0.523	0.761	0.593	−0.168***	0.761	0.761
Maximum firm size	14.746	15.512	0.766***	14.746	14.746	13.672	15.512	1.840***	13.672	13.672
Maximum firm profitability	−0.027	0.055	0.083***	−0.027	−0.027	0.065	0.055	−0.010	0.065	0.065
Large component	0.914	0.933	0.020	0.914	0.914	1.000	0.933	−0.067**	1.000	0.999
Small board size sector	0.543	0.547	0.004	0.543	0.543	0.934	0.547	−0.387***	0.934	0.934
Country’s stock market size (%)	4.349	4.365	0.016	4.349	4.349	4.349	4.365	0.016	4.349	4.348
Observations	151	19065	19216	151	6318	113	19065	19178	113	12277

S6.1 Appendix Difference in coefficients before and after the passage of affirmative action programs in entropy balanced matching analysis

Fig 5a in the manuscript shows that on average there is a positive change in the coefficient *Woman director* × *Affirmative action program* × *Eigenvector centrality* when comparing the pre-quota and post-quota settings. Fig 5b in the manuscript shows for most of the cases there is no change in the coefficient *Woman director* × *Affirmative action program* × *Eigenvector centrality* when comparing the pre-target and post-target settings. When there is a change, however, it tends to be a negative one. To determine whether the changes in the coefficient *Woman director* × *Affirmative action program* × *Eigenvector centrality* reported in both Fig 5a and Fig 5b are statistically significant, we apply a two-sample t-test –assuming equal variances– and summarize our findings in S9 Table. Panel A in S9 Table contains the results for binding gender quotas and Panel B contains the results for non-binding gender targets.

S9 Table. Ordinary least squares regression results for the number of board positions matched directors have depending on their gender and network position before and after the passage of affirmative action programs.
All coefficients correspond to the interaction term between the type of affirmative action program, woman director and eigenvector centrality. The **Coefficient Difference** column reports the difference in the coefficients pre- and post-passage of affirmative action programs and their associated significance levels. * $p < 0.10$, ** $p < 0.05$, *** $p < 0.010$

Panel A: Binding gender quota with entropy balanced sample			
	Pre-Quota Coefficient	Post-Quota Coefficient	Coefficient Difference
Belgium	0.272	0.322	0.049
France	0.013	0.459***	0.446***
Germany	−0.063	−0.201	−0.138
Italy	0.006	0.615**	0.609**
Norway	−11.094***	1.205***	0.753***
Portugal	−0.476***	−128.322	−127.846***
**Panel B: Non-binding gender target with entropy balanced sample**			
	Pre-Target Coefficient	Post-Target Coefficient	Coefficient Difference
Austria	−0.413	−0.434	−0.021
Denmark	0.435	−0.134	−0.568
Finland	1.518	−0.340***	−1.858***
Iceland	−14.814	−180.213**	−165.399***
Luxembourg	3.098	0.224***	−2.874***
Netherlands	0.289	0.441	0.153
Poland	−0.126	−0.087	0.040
Spain	0.290	0.046	−0.244
Sweden	1.538***	1.994***	0.456
United Kingdom	−0.120	−0.101	0.019

For the binding gender quota setting, we reject the hypothesis that the coefficients are the same pre-quota and post-quota in four out of the six cases (see Panel A in S9 Table

). With the exception of Portugal –for which we capture only one year in the post-quota period– all the significant changes in coefficients are positive. Due to a particularly small sample of treated cases in Norway pre-quota and Portugal post-quota (see S7 Table), the estimated coefficients are large in magnitude and have a higher uncertainty resulting in wider confidence intervals. For this reason, Norway is excluded from Fig 5(a) in the pre-quota setting, and Portugal is excluded from Fig 5(a) in the post-quota setting .

For the non-binding gender target setting, we reject the hypothesis that the coefficients are the same pre-target and post-target in only three out of the ten cases (see Panel B in S9 Table). In contrast to the binding gender quota setting, the significant changes in coefficients are negative. Furthermore, due to a particularly small sample of treated cases in Iceland (see S8 Table), the estimated coefficient is large in magnitude and has a higher uncertainty resulting in wider confidence intervals. For this reason, Iceland is excluded from Fig 5(b).
