# Supplementary material for: Affirmative action programs and network benefits in the number of board positions
Source: PLoS One. 2020 Aug 4;15(8):e0236721. doi: 10.1371/journal.pone.0236721 (PMC7402479; doi:10.1371/journal.pone.0236721)
Supplement: S7 Appendix — (PDF) [file pone.0236721.s007.pdf]

S7 Appendix. Additional details behind CEM Analysis

S10 Table . Descriptive statistics for pre- and post- binding gender quota analysis before and after matching samples using CEM.(a) Country: Norway

	Pre-Quota Before matching			Pre-Quota After matching		Post-Quota Before matching			Post-Quota After matching	
	Treated	Control	Coefficient Difference	Treated	Control	Treated	Control	Coefficient Difference	Treated	Control
Board experience	0.170	0.156	−0.014	0.141	0.113	0.181	0.156	−0.024***	0.178	0.138
Age	50.161	56.134	5.973***	49.925	53.423	52.183	56.134	3.951***	52.178	56.132
Graduate degree	0.574	0.593	0.018	0.603	0.691	0.619	0.593	−0.027**	0.621	0.599
Maximum firm size	13.132	15.512	2.380***	13.429	15.321	13.937	15.512	1.575***	13.941	15.485
Maximum firm profitability	−0.023	0.055	0.078***	−0.026	0.054	0.040	0.055	0.015***	0.040	0.056
Large component	0.766	0.933	0.168***	0.931	0.986	0.937	0.933	−0.004	0.940	0.963
Small board size sector	0.732	0.547	−0.184***	0.723	0.537	0.650	0.547	−0.103***	0.650	0.556
Country's stock market size (%)	3.985	4.365	0.380***	3.985	4.932	3.985	4.365	0.380***	3.985	4.351
Observations	423	19065	19488	305	582	4104	19065	23169	4091	17099

(b) Country: Italy

	Pre-Quota Before matching			Pre-Quota After matching		Post-Quota Before matching			Post-Quota After matching	
	Treated	Control	Coefficient Difference	Treated	Control	Treated	Control	Coefficient Difference	Treated	Control
Board experience	0.225	0.156	−0.069***	0.221	0.162	0.171	0.156	−0.015*	0.171	0.139
Age	57.394	56.134	−1.260***	57.396	55.396	57.919	56.134	−1.785***	57.939	56.787
Graduate degree	0.223	0.593	0.370***	0.226	0.620	0.281	0.593	0.311***	0.282	0.572
Maximum firm size	16.078	15.512	−0.566***	16.099	15.696	15.585	15.512	−0.073*	15.588	15.427
Maximum firm profitability	0.041	0.055	0.015***	0.041	0.069	0.031	0.055	0.024***	0.031	0.045
Large component	0.972	0.933	−0.038***	0.982	0.979	0.969	0.933	−0.035***	0.972	0.973
Small board size sector	0.385	0.547	0.162***	0.384	0.497	0.499	0.547	0.048***	0.498	0.577
Country's stock market size (%)	3.720	4.365	0.645***	3.720	4.538	3.720	4.365	0.645***	3.720	4.290
Observations	4079	19065	23144	4012	7017	4792	19065	23857	4773	10802

(c) Country: France

	Pre-Quota Before matching			Pre-Quota After matching		Post-Quota Before matching			Post-Quota After matching	
	Treated	Control	Coefficient Difference	Treated	Control	Treated	Control	Coefficient Difference	Treated	Control
Board experience	0.178	0.156	−0.022***	0.171	0.166	0.162	0.156	−0.006	0.159	0.152
Age	55.714	56.134	0.420**	55.769	55.366	57.156	56.134	−1.022***	57.138	56.733
Graduate degree	0.430	0.593	0.163***	0.434	0.627	0.478	0.593	0.115***	0.478	0.575
Maximum firm size	15.173	15.512	0.339***	15.219	15.613	14.577	15.512	0.935***	14.601	15.424
Maximum firm profitability	0.046	0.055	0.009***	0.046	0.070	0.018	0.055	0.037***	0.018	0.046
Large component	0.913	0.933	0.021***	0.926	0.948	0.925	0.933	0.008**	0.933	0.957
Small board size sector	0.638	0.547	−0.090***	0.638	0.505	0.701	0.547	−0.154***	0.701	0.580
Country's stock market size (%)	4.379	4.365	−0.014	4.379	4.517	4.379	4.365	−0.014	4.379	4.276
Observations	9149	19065	28214	8954	7351	11234	19065	30299	11095	11300

(d) Country: Belgium

	Pre-Quota Before matching			Pre-Quota After matching		Post-Quota Before matching			Post-Quota After matching	
	Treated	Control	Coefficient Difference	Treated	Control	Treated	Control	Coefficient Difference	Treated	Control
Board experience	0.193	0.156	−0.037***	0.189	0.147	0.180	0.156	−0.024**	0.181	0.129
Age	53.577	56.134	2.557***	53.584	55.355	56.458	56.134	−0.324	56.431	56.825
Graduate degree	0.643	0.593	−0.050***	0.648	0.640	0.676	0.593	−0.084***	0.676	0.584
Maximum firm size	14.593	15.512	0.919***	14.610	15.594	14.612	15.512	0.900***	14.620	15.427
Maximum firm profitability	0.059	0.055	−0.004	0.060	0.067	0.043	0.055	0.012***	0.043	0.046
Large component	0.937	0.933	−0.003	0.940	0.979	0.976	0.933	−0.043***	0.978	0.981
Small board size sector	0.592	0.547	−0.044***	0.591	0.504	0.649	0.547	−0.102***	0.649	0.578
Country's stock market size (%)	4.205	4.365	0.160***	4.205	4.544	4.205	4.365	0.160***	4.205	4.298
Observations	2835	19065	21900	2810	6873	2756	19065	21821	2751	10651

(e) Country: Germany

	Pre-Quota Before matching			Pre-Quota After matching		Post-Quota Before matching			Post-Quota After matching	
	Treated	Control	Coefficient Difference	Treated	Control	Treated	Control	Coefficient Difference	Treated	Control
Board experience	0.143	0.156	0.014***	0.141	0.165	0.137	0.156	0.019***	0.136	0.134
Age	53.982	56.134	2.152***	53.990	55.663	55.649	56.134	0.484**	55.655	57.180
Graduate degree	0.551	0.593	0.042***	0.553	0.605	0.519	0.593	0.074***	0.521	0.576
Maximum firm size	15.329	15.512	0.183***	15.344	15.655	14.833	15.512	0.679***	14.892	15.159
Maximum firm profitability	0.044	0.055	0.011***	0.044	0.062	0.032	0.055	0.023***	0.031	0.040
Large component	0.926	0.933	0.008**	0.930	0.953	0.915	0.933	0.019***	0.932	0.961
Small board size sector	0.676	0.547	−0.128***	0.676	0.528	0.732	0.547	−0.185***	0.730	0.595
Country's stock market size (%)	3.839	4.365	0.526***	3.839	4.389	3.839	4.365	0.526***	3.839	4.347
Observations	15746	19065	34811	15665	12926	5517	19065	24582	5405	5645

(f) Country: Portugal

	Pre-Quota Before matching			Pre-Quota After matching		Post-Quota Before matching			Post-Quota After matching	
	Treated	Control	Coefficient Difference	Treated	Control	Treated	Control	Coefficient Difference	Treated	Control
Board experience	0.149	0.156	0.007	0.145	0.114	0.099	0.156	0.058*	0.089	0.048
Age	54.925	56.134	1.209***	54.938	55.832	57.759	56.134	−1.625*	57.772	57.521
Graduate degree	0.469	0.593	0.124***	0.470	0.585	0.556	0.593	0.037	0.557	0.554
Maximum firm size	15.746	15.512	−0.234***	15.757	15.756	15.213	15.512	0.299	15.256	15.282
Maximum firm profitability	0.042	0.055	0.013***	0.042	0.048	0.031	0.055	0.025**	0.030	0.019
Large component	0.943	0.933	−0.010	0.948	0.969	0.951	0.933	−0.017	0.962	0.984
Small board size sector	0.424	0.547	0.123***	0.424	0.528	0.588	0.547	−0.041	0.582	0.536
Country's stock market size (%)	3.530	4.365	0.835***	3.530	4.335	3.530	4.365	0.835***	3.530	4.444
Observations	1926	19065	20991	1916	14326	162	19065	19227	158	856

S11 Table . Descriptive statistics for pre- and post- non-binding gender target analysis before and after matching samples using CEM.(a) Country: Sweden

	Pre-Target Before matching			Pre-Target After matching		Post-Target Before matching			Post-Target After matching	
	Treated	Control	Coefficient Difference	Treated	Control	Treated	Control	Coefficient Difference	Treated	Control
Board experience	0.194	0.156	-0.038^{***}	0.187	0.155	0.193	0.156	-0.037^{***}	0.192	0.139
Age	53.848	56.134	2.286^{***}	53.830	55.371	56.276	56.134	-0.142	56.285	56.693
Graduate degree	0.677	0.593	-0.084^{***}	0.681	0.650	0.691	0.593	-0.099^{***}	0.692	0.593
Maximum firm size	13.276	15.512	2.236^{***}	13.296	15.587	13.863	15.512	1.649^{***}	13.866	15.459
Maximum firm profitability	0.046	0.055	0.009^{***}	0.047	0.072	0.051	0.055	0.005^{*}	0.051	0.046
Large component	0.966	0.933	-0.032^{***}	0.971	0.982	0.991	0.933	-0.058^{***}	0.992	0.984
Small board size sector	0.559	0.547	-0.011	0.557	0.499	0.593	0.547	-0.045^{***}	0.592	0.572
Country's stock market size (%)	4.513	4.365	-0.148^{***}	4.513	4.578	4.513	4.365	-0.148^{***}	4.513	4.309
Observations	4667	19065	23732	4610	5815	4419	19065	23484	4407	11767

(b) Country: Finland

	Pre-Target Before matching			Pre-Target After matching		Post-Target Before matching			Post-Target After matching	
	Treated	Control	Coefficient Difference	Treated	Control	Treated	Control	Coefficient Difference	Treated	Control
Board experience	0.215	0.156	-0.058^{***}	0.214	0.144	0.213	0.156	-0.057^{***}	0.213	0.125
Age	55.458	56.134	0.676	55.459	55.676	56.313	56.134	-0.179	56.316	56.795
Graduate degree	0.861	0.593	-0.268^{***}	0.865	0.814	0.861	0.593	-0.269^{***}	0.862	0.635
Maximum firm size	14.864	15.512	0.648^{***}	14.879	15.550	14.516	15.512	0.996^{***}	14.517	15.396
Maximum firm profitability	0.085	0.055	-0.029^{***}	0.085	0.075	0.059	0.055	-0.004	0.059	0.055
Large component	0.952	0.933	-0.018	0.955	0.979	0.996	0.933	-0.063^{***}	0.996	0.993
Small board size sector	0.516	0.547	0.032	0.518	0.512	0.571	0.547	-0.024^{*}	0.571	0.580
Country's stock market size (%)	4.766	4.365	-0.401^{***}	4.766	4.775	4.766	4.365	-0.401^{***}	4.766	4.316
Observations	624	19065	19689	621	2878	2150	19065	21215	2149	12637

(c) Country: Denmark

	Pre-Target Before matching			Pre-Target After matching		Post-Target Before matching			Post-Target After matching	
	Treated	Control	Coefficient Difference	Treated	Control	Treated	Control	Coefficient Difference	Treated	Control
Board experience	0.167	0.156	−0.011	0.166	0.138	0.131	0.156	0.025**	0.130	0.104
Age	54.526	56.134	1.608***	54.525	55.162	54.930	56.134	1.204***	54.948	56.636
Graduate degree	0.756	0.593	−0.163***	0.756	0.677	0.774	0.593	−0.182***	0.775	0.619
Maximum firm size	15.238	15.512	0.274***	15.239	15.702	14.989	15.512	0.523***	14.992	15.431
Maximum firm profitability	0.085	0.055	−0.029***	0.085	0.077	0.069	0.055	−0.014***	0.069	0.046
Large component	0.999	0.933	−0.066***	0.999	0.997	0.997	0.933	−0.063***	0.997	0.998
Small board size sector	0.523	0.547	0.024	0.523	0.497	0.553	0.547	−0.005	0.552	0.572
Country's stock market size (%)	4.023	4.365	0.342***	4.023	4.587	4.023	4.365	0.342***	4.023	4.321
Observations	1175	19065	20240	1174	5239	1796	19065	20861	1794	10522

(d) Country: Poland

	Pre-Target Before matching			Pre-Target After matching		Post-Target Before matching			Post-Target After matching	
	Treated	Control	Coefficient Difference	Treated	Control	Treated	Control	Coefficient Difference	Treated	Control
Board experience	0.088	0.156	0.068***	0.086	0.086	0.105	0.156	0.051***	0.102	0.103
Age	49.704	56.134	6.430***	49.738	53.710	51.660	56.134	4.474***	51.686	56.031
Graduate degree	0.584	0.593	0.009	0.583	0.657	0.634	0.593	−0.042**	0.634	0.613
Maximum firm size	15.630	15.512	−0.118	15.628	15.723	15.569	15.512	−0.057	15.578	15.528
Maximum firm profitability	0.067	0.055	−0.012*	0.067	0.077	0.042	0.055	0.014***	0.042	0.044
Large component	0.951	0.933	−0.017	0.953	0.984	0.980	0.933	−0.047***	0.986	0.984
Small board size sector	0.287	0.547	0.261***	0.287	0.467	0.376	0.547	0.171***	0.377	0.566
Country's stock market size (%)	3.499	4.365	0.866***	3.499	4.509	3.499	4.365	0.866***	3.499	4.290
Observations	466	19065	19531	465	4171	1365	19065	20430	1357	10244

(e) Country: Austria

	Pre-Target Before matching			Pre-Target After matching		Post-Target Before matching			Post-Target After matching	
	Treated	Control	Coefficient Difference	Treated	Control	Treated	Control	Coefficient Difference	Treated	Control
Board experience	0.191	0.156	−0.035*	0.189	0.118	0.132	0.156	0.024***	0.132	0.127
Age	53.990	56.134	2.144***	53.972	55.078	54.810	56.134	1.324***	54.780	56.191
Graduate degree	0.575	0.593	0.018	0.578	0.683	0.544	0.593	0.049***	0.543	0.586
Maximum firm size	16.003	15.512	−0.491***	16.021	15.944	15.551	15.512	−0.039	15.563	15.591
Maximum firm profitability	0.043	0.055	0.012**	0.043	0.067	0.043	0.055	0.013***	0.043	0.053
Large component	0.975	0.933	−0.042***	0.977	0.985	0.926	0.933	0.007	0.929	0.961
Small board size sector	0.558	0.547	−0.011	0.559	0.474	0.619	0.547	−0.072***	0.618	0.558
Country's stock market size (%)	3.362	4.365	1.003***	3.362	4.694	3.362	4.365	1.003***	3.362	4.282
Observations	602	19065	19667	599	2887	2983	19065	22048	2972	13777

(f) Country: Netherlands

	Pre-Target Before matching			Pre-Target After matching		Post-Target Before matching			Post-Target After matching	
	Treated	Control	Coefficient Difference	Treated	Control	Treated	Control	Coefficient Difference	Treated	Control
Board experience	0.171	0.156	−0.015	0.157	0.161	0.131	0.156	0.025***	0.131	0.142
Age	55.543	56.134	0.591**	55.546	55.527	57.020	56.134	−0.886***	57.002	56.375
Graduate degree	0.598	0.593	−0.005	0.608	0.663	0.629	0.593	−0.036***	0.629	0.588
Maximum firm size	14.800	15.512	0.712***	14.835	15.566	14.879	15.512	0.633***	14.883	15.518
Maximum firm profitability	0.041	0.055	0.014***	0.042	0.071	0.038	0.055	0.017***	0.038	0.050
Large component	0.975	0.933	−0.042***	0.982	0.967	0.959	0.933	−0.025***	0.961	0.968
Small board size sector	0.581	0.547	−0.034**	0.581	0.494	0.651	0.547	−0.104***	0.651	0.561
Country's stock market size (%)	4.480	4.365	−0.115***	4.480	4.729	4.480	4.365	−0.115***	4.480	4.291
Observations	2234	19065	21299	2185	3866	4122	19065	23187	4111	14225

(g) Country: Spain

	Pre-Target Before matching			Pre-Target After matching		Post-Target Before matching			Post-Target After matching	
	Treated	Control	Coefficient Difference	Treated	Control	Treated	Control	Coefficient Difference	Treated	Control
Board experience	0.227	0.156	−0.071***	0.223	0.169	0.178	0.156	−0.021***	0.177	0.147
Age	56.543	56.134	−0.409	56.579	55.850	58.619	56.134	−2.485***	58.610	56.256
Graduate degree	0.429	0.593	0.164***	0.435	0.675	0.490	0.593	0.103***	0.490	0.577
Maximum firm size	16.309	15.512	−0.797***	16.343	15.762	16.134	15.512	−0.622***	16.138	15.534
Maximum firm profitability	0.060	0.055	−0.005*	0.061	0.067	0.045	0.055	0.010***	0.045	0.053
Large component	0.972	0.933	−0.038***	0.984	0.988	0.958	0.933	−0.025***	0.960	0.955
Small board size sector	0.496	0.547	0.051***	0.503	0.486	0.574	0.547	−0.027***	0.574	0.556
Country’s stock market size (%)	4.348	4.365	0.017	4.348	4.829	4.348	4.365	0.017	4.348	4.283
Observations	1835	19065	20900	1796	2791	5112	19065	24177	5101	15446

(h) Country: United Kingdom

	Pre-Target Before matching			Pre-Target After matching		Post-Target Before matching			Post-Target After matching	
	Treated	Control	Coefficient Difference	Treated	Control	Treated	Control	Coefficient Difference	Treated	Control
Board experience	0.080	0.156	0.077***	0.077	0.088	0.096	0.156	0.061***	0.093	0.070
Age	56.806	56.134	−0.672*	56.805	55.844	58.666	56.134	−2.532***	58.684	57.259
Graduate degree	0.503	0.593	0.089***	0.504	0.615	0.477	0.593	0.116***	0.478	0.581
Maximum firm size	13.644	15.512	1.868***	13.654	15.600	13.780	15.512	1.732***	13.785	15.356
Maximum firm profitability	0.021	0.055	0.034***	0.022	0.068	0.040	0.055	0.016***	0.039	0.046
Large component	0.945	0.933	−0.012	0.948	0.983	0.993	0.933	−0.060***	0.994	0.995
Small board size sector	0.636	0.547	−0.089***	0.635	0.517	0.678	0.547	−0.130***	0.677	0.596
Country’s stock market size (%)	4.705	4.365	−0.340***	4.705	4.508	4.705	4.365	−0.340***	4.705	4.305
Observations	1570	19065	20635	1562	7296	1347	19065	20412	1343	8621

(i) Country: Luxembourg

	Pre-Target Before matching			Pre-Target After matching		Post-Target Before matching			Post-Target After matching	
	Treated	Control	Coefficient Difference	Treated	Control	Treated	Control	Coefficient Difference	Treated	Control
Board experience	0.141	0.156	0.015	0.140	0.119	0.078	0.156	0.078***	0.077	0.066
Age	53.319	56.134	2.815***	53.378	54.843	57.371	56.134	−1.237***	57.497	56.449
Graduate degree	0.607	0.593	−0.014	0.614	0.660	0.592	0.593	0.000	0.589	0.580
Maximum firm size	14.936	15.512	0.576***	14.961	15.114	14.486	15.512	1.026***	14.492	15.329
Maximum firm profitability	0.067	0.055	−0.012*	0.067	0.096	0.039	0.055	0.016***	0.039	0.046
Large component	0.861	0.933	0.073***	0.882	0.971	0.902	0.933	0.032***	0.914	0.968
Small board size sector	0.568	0.547	−0.021	0.557	0.585	0.674	0.547	−0.127***	0.671	0.584
Country's stock market size (%)	4.833	4.365	−0.469***	4.833	4.659	4.833	4.365	−0.469***	4.833	4.278
Observations	417	19065	19482	407	2849	1310	19065	20375	1292	11628

(j) Country: Iceland

	Pre-Target Before matching			Pre-Target After matching		Post-Target Before matching			Post-Target After matching	
	Treated	Control	Coefficient Difference	Treated	Control	Treated	Control	Coefficient Difference	Treated	Control
Board experience	0.146	0.156	0.011	0.141	0.106	0.062	0.156	0.094**	0.062	0.021
Age	52.748	56.134	3.386***	52.812	52.936	54.858	56.134	1.276	54.858	55.863
Graduate degree	0.523	0.593	0.069	0.523	0.543	0.761	0.593	−0.168***	0.761	0.711
Maximum firm size	14.746	15.512	0.766***	14.749	15.816	13.672	15.512	1.840***	13.672	13.926
Maximum firm profitability	−0.027	0.055	0.083***	−0.028	0.046	0.065	0.055	−0.010	0.065	0.089
Large component	0.914	0.933	0.020	0.926	0.986	1.000	0.933	−0.067**	1.000	1.000
Small board size sector	0.543	0.547	0.004	0.542	0.414	0.934	0.547	−0.387***	0.934	0.699
Country's stock market size (%)	4.349	4.365	0.016	4.349	4.405	4.349	4.365	0.016	4.349	4.473
Observations	151	19065	19216	149	1493	113	19065	19178	113	2187

S7.1 Appendix Statistical differences in coefficient estimates on matched samples with CEM.

S12 Table. Ordinary least squares regression results for the number of board positions matched directors have depending on their gender and network position before and after the passage of affirmative action programs. All coefficients correspond to the interaction term between the type of affirmative action program, woman director and eigenvector centrality. The **Coefficient Difference** column reports the difference in the coefficients pre- and post-passage of affirmative action programs and their associated significance levels. * $p < 0.10$, ** $p < 0.05$, *** $p < 0.010$

Panel A: Binding gender quota with CEM sample			
	Pre-Quota Coefficient	Post-Quota Coefficient	Coefficient Difference
Belgium	−0.215	0.526	0.741**
France	−0.338***	0.469***	0.807***
Germany	−0.249**	−0.348*	−0.098
Italy	0.058	1.070***	1.012***
Norway	−6.430	1.006***	7.437***
Portugal	−0.746***	−13.616	−12.870
**Panel B: Non-binding gender target with CEM sample**			
	Pre-Target Coefficient	Post-Target Coefficient	Coefficient Difference
Austria	−0.191	−0.468**	−0.277
Denmark	−0.632	−0.403*	0.229
Finland	0.500	−0.333*	−0.833
Iceland	−3.608	−5.011	−1.403
Luxembourg	1.240	0.119	−1.122
Netherlands	0.550*	0.411***	−0.138
Poland	−0.436	0.086	0.522
Spain	0.708	−0.255**	−0.962***
Sweden	1.393***	1.994***	0.601***
United Kingdom	−0.023	−0.172	−0.149
