# Supplementary material for: Affirmative action programs and network benefits in the number of board positions
Source: PLoS One. 2020 Aug 4;15(8):e0236721. doi: 10.1371/journal.pone.0236721 (PMC7402479; doi:10.1371/journal.pone.0236721)
Supplement: S8 Appendix — [27]. (PDF) [file pone.0236721.s008.pdf]

S8 Appendix. Additional details behind the golden skirts and power inequality analyses

To determine whether golden skirts have emerged in Europe, we focus on whether women directors, as opposed to men directors, tend to hold the largest number of board positions. To determine whether the passage of quotas is associated with power inequality among directors in Europe, we evaluate whether the social capital of directors has become more dissimilar after the passage of quotas and whether the maximum level of social capital of directors has increased largely after the passage of quotas. To carry out these analyses, we use a sub-sample of 56,810 director-year observations consisting of 11,381 unique directors in the six European countries that have implemented binding gender quotas. The analyses are based on [27] which we use as a baseline study since it is one of the seminal studies addressing the emergence of the golden skirts, and power inequality among directors in Norway. It should be

pointed out, however, that the nature of our study is different from [27] in several ways. Our study focuses on the network benefits directors extract in terms of board positions, encompasses a larger data set of directors from all European countries that have implemented binding gender quotas as of 2017, uses more comprehensive information to construct director networks, and it captures a director's social capital with eigenvector centrality as opposed to betweenness centrality. Importantly, our social capital measure reflects the degree to which directors have relations with important members of the network. While, betweenness centrality, the social capital measure used by [27], captures the degree to which directors have a brokerage position in a network.

S13 and S14 Tables summarize the findings. From S13 Table , we note two things. First, we observe an increase in the proportion of directors who are women after the passage of quotas (from 5.61% to 22.39%). Second, we observe a slight disproportional overrepresentation of women directors holding two or more board positions (25.19% vs. 22.39%), three or more board positions (27.23% vs. 22.39%), four or more board positions (25.26% vs. 22.39%), and six or more board positions (22.64 vs. 22.39%) –after the passage of quotas–; however, women directors are slightly underrepresented among directors with five or more board positions (22.64% vs. 22.39%), and fully underrepresented among directors with seven or more board positions (0.00% vs. 22.39%). Since we do not observe a strong overrepresentation of women directors holding many board positions, especially when considering five or more board positions, our findings suggest the golden skirts phenomenon in Europe is not as strong as the one documented in Norway. Rather than observing only a small set of women directors holding most board positions, we also see a larger group of women directors holding board positions.

Panel A includes this comparison for the entire sample of directors in countries that have passed a binding gender quotas. Panel B includes this comparison for the sample of women directors in countries that have passed a binding gender quotas. Panel C includes this comparison for the sample of men directors countries that have passed a binding gender quotas. Prominent directors are those with more than one board position [27].

S13 Table . Distribution of directors and the number of board positions held before and after the passage of binding gender quotas.

Panel A: Percent distribution of directors across number of board positions

Number of board positions	Before quota: Men directors	Before quota: Women directors	Total	After quota: Men directors	After quota: Women directors	Total
1	74.17	78.87	74.43	77.49	73.71	76.64
2	16.73	13.48	16.55	15.73	17.49	16.13
3	5.64	3.72	5.53	4.41	6.01	4.77
4	2.25	3.13	2.30	1.60	2.14	1.72
5	0.73	0.59	0.72	0.55	0.41	0.52
6	0.35	0.22	0.34	0.14	0.15	0.14
7	0.12	0.00	0.12	0.07	0.08	0.07
8	0.02	0.00	0.02	0.02	0.00	0.02
Total	31,182	1,855	33,037	18,451	5,322	23,773
%	94.39	5.61	100.00	77.61	22.39	100.00

Panel B: Percent and number of women directors across cumulative number of board positions

Number of board positions	Before quota: Women directors %	Before quota: Women directors #	All directors #	After quota: Women directors %	After quota: Women directors #	All directors #
2 or more	4.64	392	8,447	25.19	1,399	5,553
3 or more	4.76	142	2,981	27.23	468	1,719
4 or more	6.33	73	1,154	25.26	148	586
5 or more	3.80	15	395	19.21	34	177
6 or more	2.56	4	156	22.64	12	53
7 or more	0.00	0	44	0.00	0	20

S14 Table . Comparison of maximum, average values for the number of board positions directors have before and after quotas, as well as their network centralities.
Panel A includes this comparison for the entire sample of directors in countries that have passed a binding gender quotas. Panel B includes this comparison for the sample of women directors in countries that have passed a binding gender quotas. Panel C includes this comparison for the sample of men directors countries that have passed a binding gender quotas. Prominent directors are those with more than one board position [27].

	Directors in countries with binding gender quotas: Before quota	Directors in countries with binding gender quotas: After quota	Prominent directors in countries with binding gender quotas: Before quota	Prominent directors in countries with binding gender quotas: After quota
**Panel A: All directors**				
Max[Number of board positions]	8.00	8.00	8.00	8.00
Avg[Number of board positions]	1.40	1.34	2.56	2.46
SD[Number of board positions]	0.83	0.74	0.93	0.82
Max[Eigenvector centrality]	100	100	100	100
Avg[Eigenvector centrality]	2.57	0.29	5.67	0.64
SD[Eigenvector centrality]	7.50	2.53	12.49	5.03
Count[Directors]	7387	7377	2001	1756
**Panel B: Women directors**				
Max[Number of board positions]	6.00	7.00	6	7
Avg[Number of board positions]	1.34	1.39	2.60	2.50
SD[Number of board positions]	0.77	0.77	0.91	0.80
Max[Eigenvector centrality]	85.11	75.41	85.11	75.41
Avg[Eigenvector centrality]	2.92	0.17	6.17	0.33
SD[Eigenvector centrality]	8.62	1.75	14.37	3.19
Count[Directors]	515 (6.97%)	1662 (22.53%)	125 (6.25%)	457 (26.03%)
**Panel C: Men directors**				
Max[Number of board positions]	8.00	8.00	8.00	8.00
Avg[Number of board positions]	1.40	1.33	2.56	2.46
SD[Number of board positions]	0.83	0.73	0.93	0.83
Max[Eigenvector centrality]	100	100	100	100
Avg[Eigenvector centrality]	2.55	0.25	5.64	0.75
SD[Eigenvector centrality]	7.43	2.71	12.39	5.51
Count[Directors]	6872 (93.03%)	5715 (77.47%)	1876 (93.8%)	1299 (73.97%)
